# Genotyping of *Bacillus cereus* Strains by Microarray-Based Resequencing

**DOI:** 10.1371/journal.pone.0002513

**Published:** 2008-07-02

**Authors:** Michael E. Zwick, Maureen P. Kiley, Andrew C. Stewart, Alfred Mateczun, Timothy D. Read

**Affiliations:** 1 Biological Defense Research Directorate, Naval Medical Research Center, Silver Spring, Maryland, United States of America; 2 Department of Human Genetics, Emory University School of Medicine, Atlanta, Georgia, United States of America; University of British Columbia, Canada

## Abstract

The ability to distinguish microbial pathogens from closely related but nonpathogenic strains is key to understanding the population biology of these organisms. In this regard, *Bacillus anthracis*, the bacterium that causes inhalational anthrax, is of interest because it is closely related and often difficult to distinguish from other members of the *B. cereus* group that can cause diverse diseases. We employed custom-designed resequencing arrays (RAs) based on the genome sequence of *Bacillus anthracis* to generate 422 kb of genomic sequence from a panel of 41 *Bacillus cereus* sensu lato strains. Here we show that RAs represent a “one reaction” genotyping technology with the ability to discriminate between highly similar *B. anthracis* isolates and more divergent strains of the *B. cereus* s.l. Clade 1. Our data show that RAs can be an efficient genotyping technology for pre-screening the genetic diversity of large strain collections to selected the best candidates for whole genome sequencing.

## Introduction

The accurate genetic characterization of microbial pathogens and closely related non-pathogenic strains is a central challenge of contemporary microbiology. Since the 2001 bioterrorist attacks in the United States, the highly virulent gram-positive endospore-forming bacterium *Bacillus anthracis* has been the subject of intense research. *B. anthracis* is a recently emerged lineage in the polyphyletic *B. cereus* sensu lato group [1]–[3]. Molecular clock estimates suggest that all *B. anthracis* strains shared a common ancestor 13,000–26,000 years ago [4]. Several high-resolution technologies have been applied to subtype strains of the *B. anthracis* classes, including the use of MLVA (multi-locus variable number of tandem repeat analysis) [5], [6], “canonical” single nucleotide polymorphism (SNP) typing [7], microarrays [8] and even whole-genome sequencing [9]. For separating *B. anthracis* from the very closely related *B. cereus* s. l. “species”, there are numerous methods available that have a slightly less sensitive level of genetic resolution, including amplified fragment length polymorphism (AFLP) analysis, and multi-locus sequence typing (MLST) [3], [10]–[15].

A particular advantage of partial or complete genome sequencing technologies (including MLST schemes) is their ability to detect both common and rare genetic variants within populations. Population genomic models frequently assume that genetic variation is detected randomly with respect to population frequency, an assumption that is violated in studies focusing solely on common (>5% frequency) variation [4]. Furthermore, although typing schemes based upon common variation are quite useful, it may often happen that rare variants are more informative for tracking the history of specific strains, as might be required in an epidemiological outbreak study. Ever increasing amounts of genome sequence may pose significant challenges in phylogenetic reconstruction of bacterial strains because of potential conflicts between gene content trees and species trees in some taxonomic groups [16], [17]. Nevertheless, it seems clear that methods that can generate greater quantities of genome sequence at lower costs are likely to be increasingly useful for characterizing pathogenic strains.

Custom-designed microarrays, termed resequencing arrays (RAs), are a highly parallel emerging technology with a rapidly diminishing cost basis that can be used to perform DNA sequencing by hybridization [18]–[21]. One potential limitation of this method of DNA sequencing is that, because the RA design is based upon a known reference sequence, strains with a higher level of nucleotide divergence to the template would be expected to generate less data than more closely related strains. Furthermore, novel DNA sequences introduced by insertion will not be detected directly, although their presence can be inferred by disruptions in normal patterns of hybridization. As others have shown, some of these technical challenges can be overcome by designing RAs based upon multiple reference sequences [20], [21].

In a previous report [19], we used an Affymetrix Inc. 18-micron feature resequencing array with a design based on ∼29 kb of the *B. anthracis* Ames genome [22] to resequence 56 *B. anthracis* strains. Here we report the results of a set of experiments using RAs of the same design to obtain the sequence from a collection of 41 diverse *Bacillus* strains. The genomic sequences were then processed through a standard phylogenetic program pipeline to obtain phylogenetic trees. These trees were then compared directly with trees obtained from multi-locus sequencing typing (MLST) of the identical strains. The results of this study suggest that *B. anthracis* RAs can be valuable for genotyping “Clade 1” [10] *B. cereus* strains: a lineage found to contain several strains of unusual virulence in recent years.

## Results

A diverse collection of 41 Bacillus strains (Table 1) were resequenced using a custom-designed RA based upon the *B. anthracis Ames* reference sequence, which we had used previously to genotype a collection of 56 *B. anthracis* strains ([19], Table S1). The genomic regions interrogated by the RA in all 41 strains for this study included 16,464 bp from the Ames chromosome, 6701 bp from plasmid pXO1 and 6677 bp from plasmid pXO2 (Tables S2 and S3). The ABACUS algorithm uses a maximum likelihood model to determine the most likely genotype and provides a log_10_ quality score (QS) for each base call [18]. Only bases exceeding the quality score threshold are called; results are summarized in Table S4.

**Table 1:** *Bacillus* strains resequenced in this study.

Strain ID	Species ID	Strain Description	MLST Serotype
BAN_001	*B. anthracis*	Sterne	1
BAN_002	*B. anthracis*	Sterne	1
BAN_003	*B. anthracis*	Ames	-
BAN_004	*B. anthracis*	Isolated Etosha National Park Namibia	1
BTU_001	*B. thuringiensis*	Serotype 11a11c; Dulmage H.; produces toxin Cyt2Aa1	109
BTU_002	*B. thuringiensis*	Berliner; isolated tissue Mediterranean flour moth	10
BTU_003	*B. thuringiensis*	Berliner; mutant selected from wild type, soil isolate	-
BTU_004	*B. thuringiensis*	-	171
BTU_005	*B. thuringiensis*	Wild type isolate Serotype; NRRL-B4039	10
BCE_001	*B. cereus*	Bacillus Genetic Stock Center; wild type isolate T	29
BCE_002	*B. cereus*	Isolated fried rice; FHL 4746	26
BCE_003	*B. cereus*	Emetic; isolated chicken korma; other name F 3080 B/87	26
BCE_004	*B. cereus*	Emetic; isolated boiled rice; other name FHL 3942	26
BCE_005	*B. cereus*	Emetic; isolated human vomit; other name SMR 178, FHL 4810	26
BCE_006	*B. cereus*	Other name 5893	26
BCE_007	*B. cereus*	-	78
BCE_008	*B. cereus*	-	164
BCE_012	*B. cereus*	Isolated milk	393
BCE_013	*B. cereus*	Other name Bonde 354; marine (North Sea)	30*
BCE_014	*B. cereus*	Egg fried rice. D&V. 2 ill	392
BCE_015	*B. cereus*	Serogroup AA. isolated from vomit 6h after Chinese meal	181
BCE_016	*B. cereus*	FEW. Serogroup G	395
BCE_017	*B. cereus*	-	389
BCE_018	*B. cereus*	Leg swab. Serogroup (17, V).	391
BCE_019	*B. cereus*	Neutropenia in child. Serogroup 3	144
BCE_022	*B. cereus*	Milk products, choc UHT milk; other name BN-1	44
BCE_023	*B. cereus*	Mineral pigment Kaolin.	39
BCE_024	*B. cereus*	Infant born very edematous; Serogroup 6; strong enterotoxin producer	394
BCE_025	*B. cereus*	Gangrene, cellulitis. Serogroup 26	18
BCE_026	*B. cereus*	Endocarditis. (NT)	145
BCE_027	*B. cereus*	ATCC 10987; Frankland and Frankland; NRS 248; Xylose-positive variant	32*
BCE_028	*B. cereus*	ATCC 14579	4*
BCE_029	*B. cereus*	Isolated Etosha National Park Namibia	1
BCE_030	*B. cereus*	Isolated Etosha National Park Namibia	1
BMG_001	*B. megaterium*	Epidural abscess.	24*
BMY_001	*B. mycoides*	NCTC 2603, NRS 935 (1940) “B. praussnitzi”	4
BMY_002	*B. mycoides*	Flugge	390
BMY_003	*B. mycoides*	Gibson 71, dust (1979); other name BGSC 6A14	187
BMY_004	*B. mycoides*	Goodfellow (1978); other name LMG 12411; Lovett 80 (A9)	222
BSU_001	*B. subtilis*	trpC2; Original code 168; Burkholder and Giles 1947	-
BSU_002	*B. subtilis*	phototrophic; other name SB491	-

**Figure 1:**
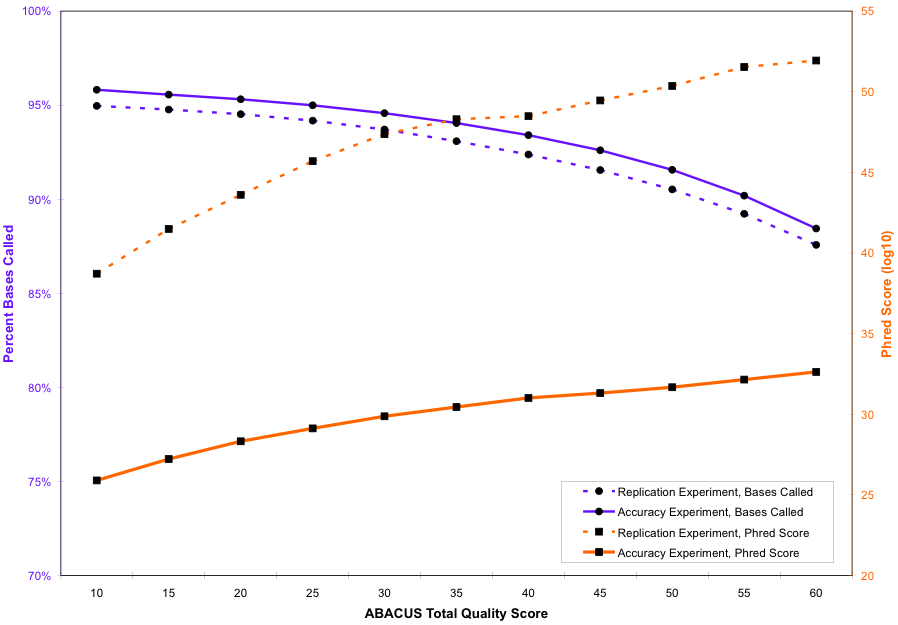
Graph shows the relationship between basecalling frequency and estimated phred score at various ABACUS total quality score thresholds. For both the replication and accuracy experiments in Zwick et al. (2005), note that as the ABACUS total quality score threshold is increased, the number of bases called is decreased, with a concomitant increase in data quality (phred score).

As expected, the pXO1 and pXO2 plasmid regions of the RA only hybridized above a background level of efficiency for basecalling (∼25%) with *B. anthracis* strains or *B. cereus* G9241, which is known to contain a plasmid with a nucleotide sequence very similar to pXO1 [23]. Therefore, we based our strain comparisons solely on the chromosomal sequences. In order to maximize the number of bases called among the divergent collection of strains resequenced, analysis with the ABACUS algorithm was performed without using the highly conservative neighborhood and sample finishing rules. These rules, when intraspecific strains are being resequenced, act to eliminate bases that may be unreliable in order to obtain the highest possible quality sequence data. Some high quality bases may also be eliminated by this analysis. Our previous work [19] has shown that RA error rates estimated from replication and independent accuracy experiments are very low at moderate ABACUS total quality scores when using neighborhood rules. To assess the effects of relaxing the neighborhood rules, we reanalyzed the RA data from the 56 *B. anthracis* strains at a variety of ABACUS Total Quality score thresholds. Figure 1 shows the relationship between the percent of bases called and the discrepancy rates (measured as a phred score, where phred = −10 log_10_ [binominal error probability]) at various ABACUS total quality score thresholds [24], [25]. Our analysis of both the replication and accuracy experiments demonstrates that very high-quality data can still be obtained, even in the absence of the neighborhood rules, which supports our method of analyzing RA data from the 41 diverse *Bacillus* strains.

**Figure 2:**
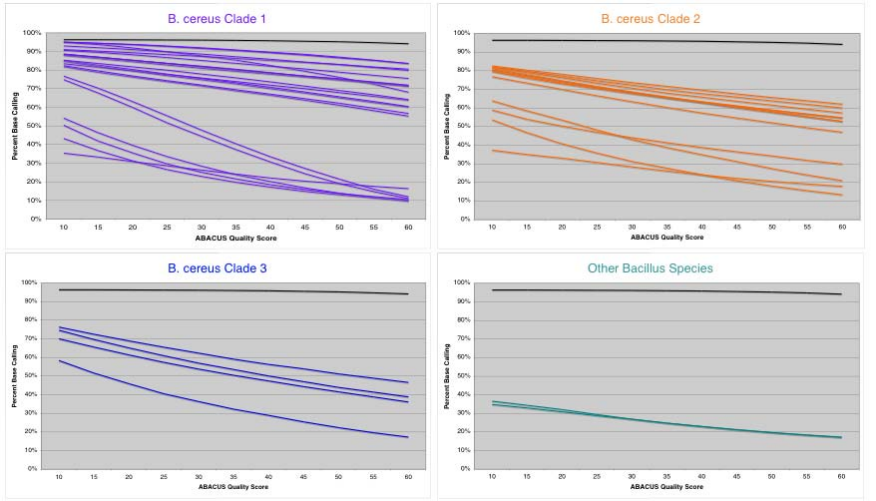
Graphs showing resequencing array (RA) percent basecalling at different ABACUS quality score (QS) thresholds. The black line in each plot shows the basecalling rate for the *B. anthracis* Ames strain that acted as the reference sequence for the design of the RA. The raw data for this figure is contained in Table S5.

Basecalling rates across the 41 strains varied widely, with those strains most closely related to *B. anthracis* (i.e. Clade 1 [10]) having the highest rates of basecalling (Figure 2). Highly diverged species, such as the *Bacillus subtilis* strains (BSU_001, BSU_002), show very low basecalling rates at all ABACUS quality scores. Low basecalling rates are expected when a strain hybridized to a chip has a high level of sequence divergence relative to the reference sequence used to design the RA. Adjusting the minimum ABACUS quality score threshold [18] from a minimum of 10 to a maximum of 60 reduces the total number of bases called for all strains (10–60, Figure 2, Table S5).

For this experiment we departed from our earlier methodology [19] by performing whole genome amplification of the target DNA prior to hybridization, instead of amplification of specific fragments by long PCR (LPCR). We chose this approach in order to eliminate the extra step of performing PCR reactions that are likely to fail when the sequence of the test strain is particularly diverged from the reference. The disadvantage of hybridizing with whole-genome DNA is the expectation that additional oligonucleotide sequences would be available to cross-hybridize, leading to spurious results. Our goal was to identify a quality score threshold and level of experimental success (as measured by basecalling rate) that enables the use of RAs for resequencing the genomic DNA of bacterial strains diverged from the reference sequence upon which the RA is based.

In order to better assess RA data quality and examine the utility of the RA sequence data for detecting phylogenetic relationships among strains, we decided to construct phylogenetic trees on a subsample of 36 strains. We independently generated MLST genotypes for the 36 strains that had not yet been typed and submitted the data to the *B. cereus* MLST database (http://pubmlst.org/bcereus/). The phylogenetic trees inferred using the MLST data (Figure S1) and RA data (Figure S2) show some striking differences in the inferred relationships among strains. In particular, the 9 *Bacillus* strains with the lowest RA base calling rates (between 9.5%–20.9%) form a clade in the RA phylogeny that is not observed in the MLST tree. Results from our own genome *B. cereus* group sequencing studies (Read et al, in preparation) and from comparison to microarray results from other species (e.g. [16]) suggest a good correlation between the MLST phylogeny and the organismal phylogeny. If we assume that the MLST phylogeny is correct, this result indicates that RAs with very low rates of basecalling will have error rates exceeding those expected from intraspecific experiments, and hence will suggest false clades.

If this is true, we can make two predictions. The first posits that raising the ABACUS quality score threshold will reduce the difference between the MLST and RA trees. Second, the RA and MLST phylogeny will show greater concordance if we restrict our analysis to interspecific strains where RA basecalling rates are elevated (>55% at a QS of 60 and >70% at an ABACUS QS threshold of 30).

**Figure 3:**
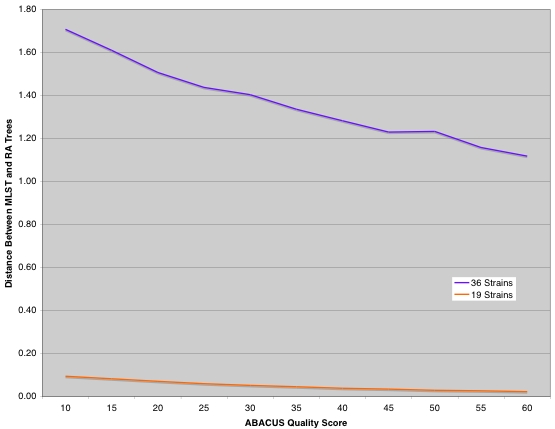
Graph shows relationship between multilocus sequence typing (MLST)/Resequencing Array(RA) tree distance and ABACUS quality score.

**Figure 4:**
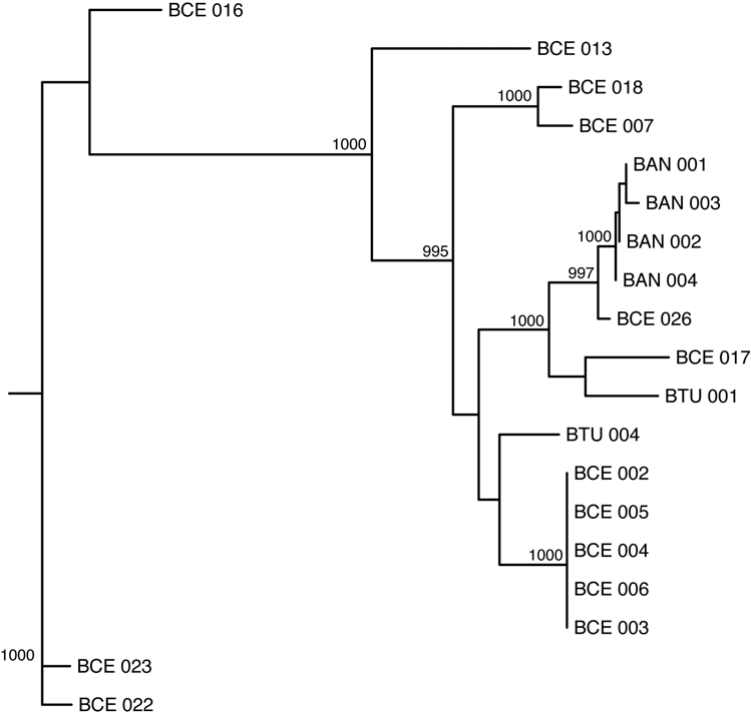
Neighbor-joining phylogenetic tree inferred using multilocus sequence typing (MLST) data for a subsample of 19 Bacillus strains. Bootstrap values for nodes with greater than 99% support (1000 replicates total) are shown.

**Figure 5:**
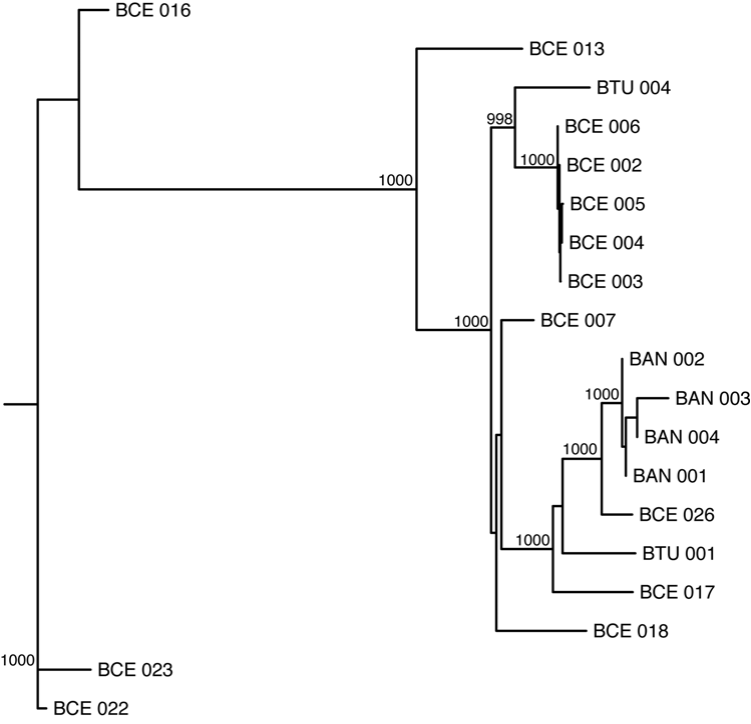
Neighbor-joining phylogenetic tree inferred using resequencing array (RA) data with a quality score threshold of 30 for a subsample of 19 *Bacillus* strains. Bootstrap values for nodes with greater than 99% support (1000 replicates total) are shown.

**Figure 6:**
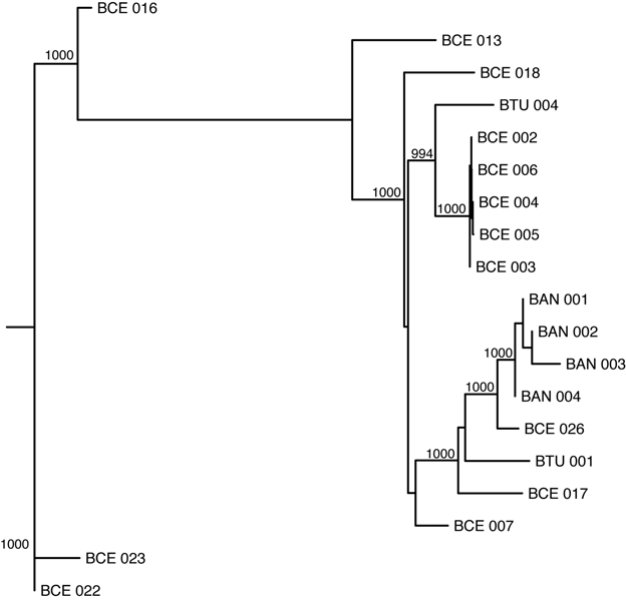
Neighbor-joining phylogenetic tree inferred using resequencing array (RA) data with a quality score threshold of 60 for a subsample of 19 Bacillus strains. Bootstrap values for nodes with greater than 99% support (1000 replicates total) are shown.

A total of 19 *Bacillus* strains had greater than 70% basecalling at an ABACUS quality score threshold of 30 (and 55% basecalling at an ABACUS QS threshold of 60). We again used MLST data from the same strains to construct phylogenetic trees. Figure 3 shows that increasing the RA data quality threshold reduces the difference between the MLST and RA trees (both 19- and 36-strain datasets) as we predicted. Furthermore, the MLST and RA (QS 30, QS 60) trees from the 19-strain dataset show remarkable agreement, in marked contrast to what was seen for the 36-strain dataset (Figures 4, 5, 6). All three trees provide very strong support for similar nodes in the trees. These data imply that accurate phylogenies can be inferred from interspecific RA data with both increased quality score thresholds and minimum basecalling rates.

## Discussion

This report demonstrates that RAs can be used effectively for genotyping strains from both within the *B. anthracis* lineage and amongst close neighbors. Microarrays offer some practical advantages over MLST for DNA sequence-based genotyping. First, potentially much more sequence can be probed on a single RA than the 2.5–3.5 kb generally used today for MLST. Furthermore, because of the limited size and complexity of a typical bacterial genome, labeled genomic DNA (in this case amplified by rolling-circle amplification) can be hybridized to the RA in a single reaction. Because generating the target DNA for hybridization is not dependent on many different PCRs, it is easy to put multiple regions of the genome on one array, including portions that may not necessarily be used for genotyping, but may point to the acquisition of key genes horizontally (in the case of *B. anthracis*, the pXO1 and pXO2 plasmids).

Current high density RAs with smaller feature sizes now allow up to 300 kb of the reference strain to be resequenced. These larger-capacity RAs mean more finely detailed mapping. For future applications of this technology, there will be a trade-off between increasing the amount of sequence on the RA for better resolution versus the lower cost of smaller chips and the potential for putting them in devices that can be transported into the clinic and the field.

From the results of this report, we conclude that a RA designed on the *B. anthracis* sequence will be most effective for genotyping strains of the clade 1 of *B. cereus* s.l. [10]. Strains of this group have a maximal nucleotide sequence divergence of ∼5% for each of the chromosomal regions represented on the RA. More distantly related strains (found in other clades *of B. cereus* s.l. and other *Bacillus* species) would be surveyed less efficiently because of their greater nucleotide divergence. Nevertheless, since the highest proportion of strains pathogenic to humans are contained within clade 1, this RA approach could be an effective tool for discriminating among these strains. Notable clade 1 strains outside *B. anthracis* include G9241 [26], which was associated with lethal fulminant pneumonia in humans, *B. cereus* strains recently reported to have killed chimpanzees in Africa [27] and the emetic food poisoning strains of *B. cereus* [28]. The latter group has certain parallels to *B. anthracis*; namely, a largely clonal population structure related to the recent acquisition of a toxin (cereulide, which causes emesis) on a large plasmid with a similar backbone to pXO1 [29]. Like *B. anthracis*, the very low level of diversity makes subtyping difficult, with most strains falling into just one MLST sequence type (ST26, [10]). Interestingly, this study shows that ST26 can be subdivided by microarray-based resequencing using the *B. anthracis* template (Figures 5 and 6).

Comparative whole genome sequencing of the *B. anthracis* attack strain using traditional Sanger chemistry at a genome sequencing center identified a number of polymorphisms relative to the reference strain [9]. This particular study concluded that genome-based analysis of microbial pathogens could provide a powerful new tool for the investigation of infectious disease outbreaks. The major drawback of its application, however, was cost. Population genomic studies of microbial pathogens and their closely related nonpathogenic relatives have come within practical reach only recently, due to dramatic reductions in sequencing costs associated with the adoption of novel DNA sequencing technologies [30]. Eventually, whole genome sequencing of very large populations of bacterial strains may be economically viable. However, for the foreseeable future, a genotyping technology that can prescreen the genetic diversity of large strain collections will be necessary to select the best candidates for whole genome sequencing. Results from this study indicate that microarray-based resequencing may be capable of fulfilling this important role.

## Materials and Methods

### Bacillus Strains Surveyed

We selected a diverse panel of 41 *Bacillus* strains from the Biological Defense Research Directorate (BDRD) collection at the Navy Medical Research Center (NMRC) for chip resequencing (see Table 1). Thirty-six of the strains were also typed by MLST using ABI sequencing [10]. These data are available through the *Bacillus cereus* MLST website (http://pubmlst.org/bcereus/).

### RA Design, Hybridization, Sequence Determination

The RA design was based upon the *B. anthracis* Ames reference sequence (5.2 Mbp, NC_003997) as previously described [19]. The chromosomal regions interrogated by the RA in all 41 strains included 16,584bp from the Ames chromosome and included the following genes: *vrrA*; DNA-directed RNA polymerase, *rpoB*; *yfhp* protein. Genomic DNA from each strain was isolated using standard protocols as previously described [19]. Target DNA for RA hybridization was obtained by performing whole genome amplification (WGA) on 100 ng of genomic DNA following the manufacturer's instructions (REPLI-g Kit from Qiagen, Valencia, CA). The typical yield was 20–30 ug per strain. The WGA DNA was then DNAse digested, biotin end-labelled, and hybridized to individual RAs overnight following established protocols [19]. Subsequent washes and stains were carried out following the RA manufacturers standard protocols (Affymetrix, Sunnyvale, CA). RAs were scanned at 570 nm, with a pixel size of 3 µ per pixel averaged over 2 scans. Genomic sequences were determined for each sample by using the ABACUS algorithm as implemented in RATools (http://www.dpgp.org) [18], [19]. Sequence finishing rules (sample number, amplicon failure, neighborhood failure) typically applied when resequencing closely related species were not applied to the raw basecalls because of the large evolutionary divergence between some of the strains.

### Phylogenetic Analysis

The PHYLIP package (3.67) was used for all phylogenetic analyses [31]. Inferred RA genome sequences for each strain were concatenated to create a single strain sequence in FASTA format. MLST genome sequences were concatenated to create a single strain sequence in FASTA format (16,464 bp total). RA and MLST sequences were converted to PHYLIP format using Clustal X [32] for subsequent analyses.

A Perl script (Phylip_neighbor_distance.pl) that called the PHYLIP program’s dnadist and neighbor modules was used to generate a distance matrix and determine a neighbor-joining (NJ) tree for both the RA and MLST datasets. A separate Perl script (Phylip_boot_distance.pl) that called the PHYLIP program’s seqboot, dnadist, neighbor and consense was used to generate 1000 replicate data sets for bootstrap analysis of the NJ trees. The PHYLIP program drawgram was used to draw the NJ trees. A Perl script (Phylip_tree_distance.pl) was used to run the PHYLIP treedist program to determine the distance between the RA and MLST trees using the Branch Score Distance [33].

## Supporting Information

Figure S1 Phylogenetic tree inferred for 36-strains with MLST data. Neighbor-joining phylogenetic tree inferred using multilocus sequence typing (MLST) data for a subsample of 36 B. cereus strains. Bootstrap values for nodes with greater than 99% support (1000 replicates total) are shown.
(0.97 MB TIF)

Figure S2 Phylogenetic tree inferred for 36-strains with RA data. Neighbor-joining phylogenetic tree inferred using resequencing array (RA) data with a quality score threshold of 30 for a subsample of 36 B. cereus strains. Bootstrap values for nodes with greater than 99% support (1000 replicates total) are shown. The purple box marks a clade of Bacillus strains with low basecalling rates. This clade is not observed in comparable MLST phylogenetic tree (Figure S1). We infer that the low RA basecalling rates for these strains resulted in their clustering together in a false clade.
(1.03 MB TIF)

Table S1 *B. anthracis* strains resequenced in Zwick, M. E. et al. (2005)
(0.06 MB PDF)

Table S2 BDRD-01 resequencing array (RA) reference sequence information.
(0.02 MB PDF)

Table S3 Reference genomic sequence used to design the BDRD-01 resequencing array (RA).
(0.03 MB PDF)

Table S4 Proportion bases called (QS 30) for each *B. anthracis* molecule
(0.04 MB PDF)

Table S5 Bacillus resequencing results.
(0.05 MB PDF)
