# Supplementary figures and images for: Genotyping of Bacillus cereus Strains by Microarray-Based Resequencing
Source: PLoS One. 2008 Jul 2;3(7):e2513. doi: 10.1371/journal.pone.0002513 (PMC2438477; doi:10.1371/journal.pone.0002513)

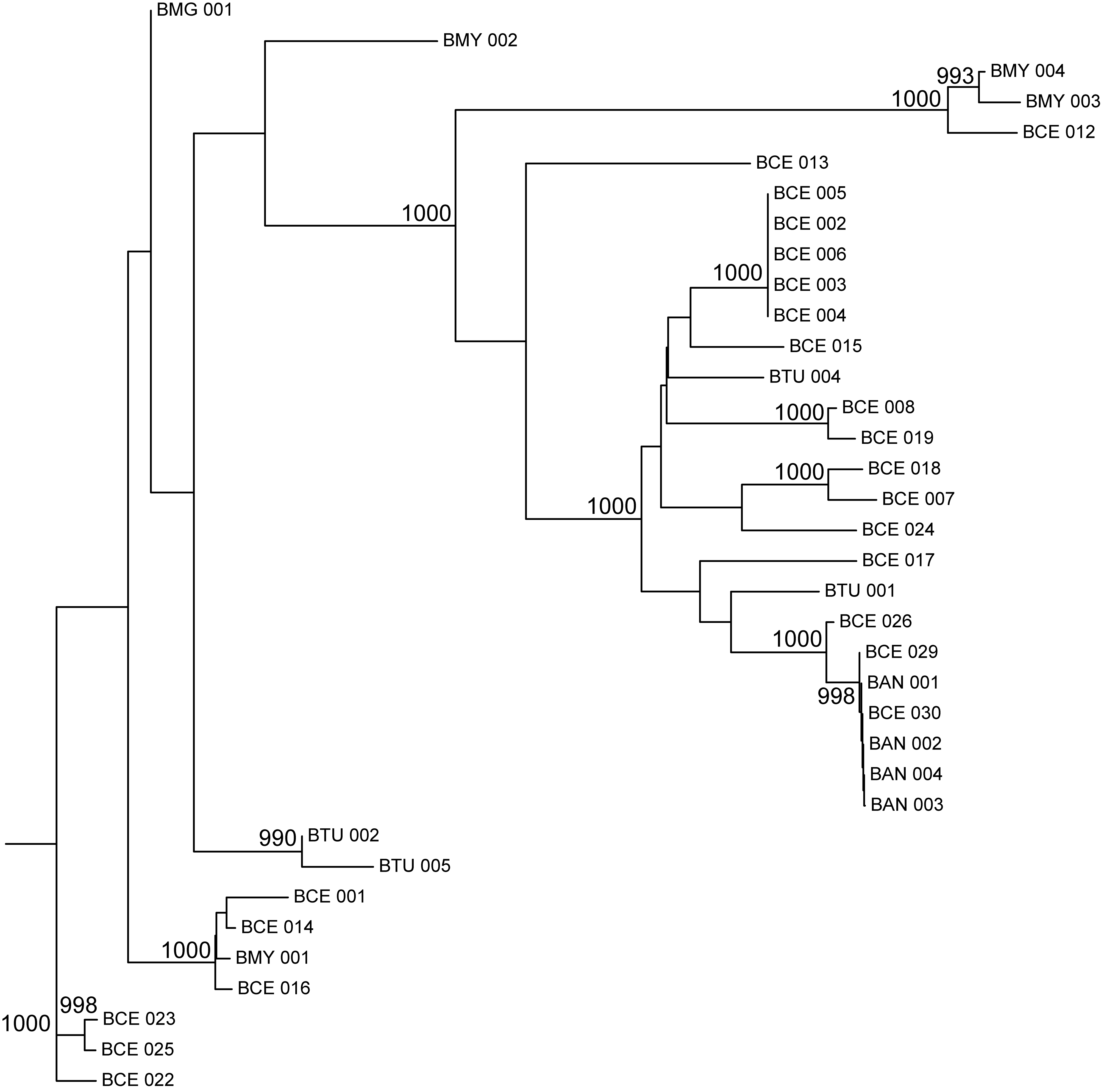

Supplement: Figure S1 — Phylogenetic tree inferred for 36-strains with MLST data. Neighbor-joining phylogenetic tree inferred using multilocus sequence typing (MLST) data for a subsample of 36 B. cereus strains. Bootstrap values for nodes with greater than 99% support (1000 replicates total) are shown. (0.97 MB TIF) [file pone.0002513.s001.tif]

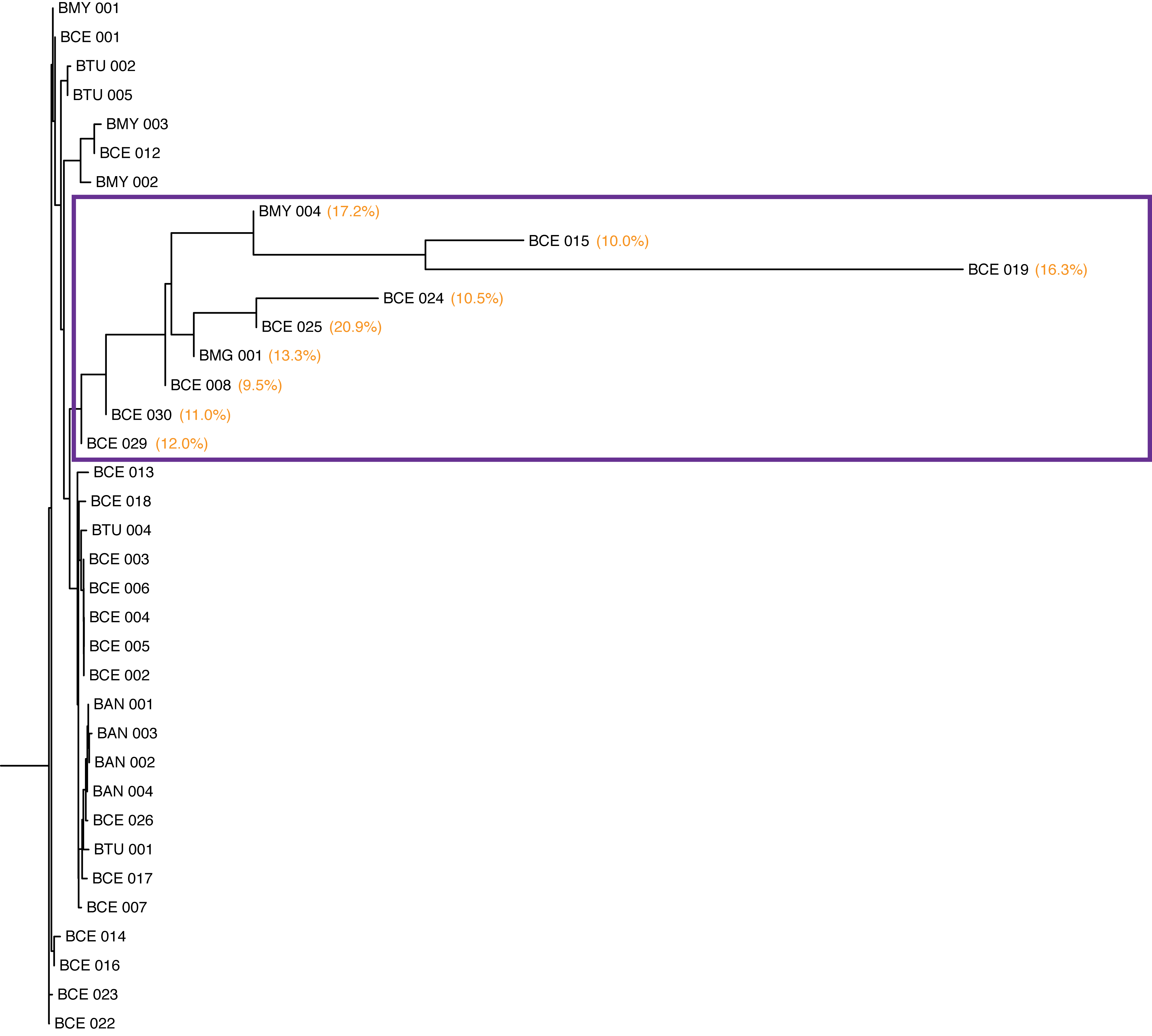

Supplement: Figure S2 — Phylogenetic tree inferred for 36-strains with RA data. Neighbor-joining phylogenetic tree inferred using resequencing array (RA) data with a quality score threshold of 30 for a subsample of 36 B. cereus strains. Bootstrap values for nodes with greater than 99% support (1000 replicates total) are shown. The purple box marks a clade of Bacillus strains with low basecalling rates. This clade is not observed in comparable MLST phylogenetic tree (Figure S1). We infer that the low RA basecalling rates for these strains resulted in their clustering together in a false clade. (1.03 MB TIF) [file pone.0002513.s002.tif]
