# Supplementary material for: Genotyping of Bacillus cereus Strains by Microarray-Based Resequencing
Source: PLoS One. 2008 Jul 2;3(7):e2513. doi: 10.1371/journal.pone.0002513 (PMC2438477; doi:10.1371/journal.pone.0002513)
Supplement: Table S2 — BDRD-01 resequencing array (RA) reference sequence information. (0.02 MB PDF) [file pone.0002513.s004.pdf]

BDRD-01 RA Fragment Name	GenBank Reference Sequence	Location of Sequence	Genomic Sequence Capable of Being Queried
nmrc_001	NC_001496	pXO1	929
nmrc_002	NC_001496	pXO1	583
nmrc_003	NC_001496	pXO1	1,496
nmrc_004	NC_001496	pXO1	1,256
nmrc_005	NC_001496	pXO1	1,807
nmrc_006	NC_002146	pXO2	747
nmrc_007	NC_002146	pXO2	5,930
nmrc_008	NC_003997	Main Chromosome	3,029
nmrc_009	NC_003997	Main Chromosome	2,082
nmrc_010	NC_003997	Main Chromosome	6,053
nmrc_011	NC_003997	Main Chromosome	2,685
nmrc_012	NC_003997	Main Chromosome	2,615
			29,212
