# Supplementary material for: Genotyping of Bacillus cereus Strains by Microarray-Based Resequencing
Source: PLoS One. 2008 Jul 2;3(7):e2513. doi: 10.1371/journal.pone.0002513 (PMC2438477; doi:10.1371/journal.pone.0002513)
Supplement: Table S3 — Reference genomic sequence used to design the BDRD-01 resequencing array (RA). (0.03 MB PDF) [file pone.0002513.s005.pdf]

>nmrc_001
ATTAAAAAATTATATGCTATGTCAATTATATTCATAGCGCGTTTTTTACCGTTATAAAAGATAAAAACTCGAAAACAGAAAAATAATATACCTTT
TATTCTATACAGCAACCTAAATATTATAATCAACTTTTCCATAGAAATTAATCCTTTTGTTATACATCTTTATTCCCTTAACATTGTCAAATTTC
AGTTATTCATTCTGGATAGTCAATAAATAGATTACGGTTATGTTAGTATTTTTTTAAAATAATAGTATTAAATAGTGGAATGCAAATGATAAATG
GGCTTTAAACAAAACTAATGAAATAATCTACAAATGGAATTTCTCCAGTTTTAGATTAAACCATACCAAAAAAATCACACTGTCAAGAAAAATGA
TAGAATCCCTACACTAATTAACATAACCAAATTGGTAGTTATAGGTAGAAACTTATTTATTTCTATAATACCATGCAAAAAACAAACTAAATATT
CTGTTCCATACTATTTTAGTAAATTATTTAGCAAGTAAATTTTGGTGTATAAACAAAGTTTATCTTAATATAAAAAATTACTTTACTTTTATACA
GATTAAAATGAAAAATTTTTTATGACAAGAAATATTGCCTTTAATTTATGAGGAAATAAGTAAAATTTTCTACATACTTTATTTTATTGTTGAAA
TGTTCACTTATAAAAAAGGAGAGATTAAATATGAATATAAAAAAAGAATTTATAAAAGTAATTAGTATGTCATGTTTAGTAACAGCAATTACTTT
GAGTGGTCCCGTCTTTATCCCCCTTGTACAGGGGGCGGGCGGTCATGGTGATGTAGGTATGCACGTAAAAGAGAAAGAGAAAAATAAAGATGAGA
ATAAGAGAAAAGATGAAGAACGAAATAAAACACAGGAAGAGCATTTAAAGGAAATCATGAAACACATTGTAAAAATAGAAGTAAAAGGGGAGGAA
GCT
>nmrc_002
ATATATATTGTGGATGGTGATATTACAAAACATATATCTTTAGAAGCATTATCTGAAGATAAGAAAAAAATAAAAGACATTTATGGGAAAGATGC
TTTATTACATGAACATTATGTATATGCAAAAGAAGGATATGAACCCGTACTTGTAATCCAATCTTCGGAAGATTATGTAGAAAATACTGAAAAGG
CACTGAACGTTTATTATGAAATAGGTAAGATATTATCAAGGGATATTTTAAGTAAAATTAATCAACCATATCAGAAATTTTTAGATGTATTAAAT
ACCATTAAAAATGCATCTGATTCAGATGGACAAGATCTTTTATTTACTAATCAGCTTAAGGAACATCCCACAGACTTTTCTGTAGAATTCTTGGA
ACAAAATAGCAATGAGGTACAAGAAGTATTTGCGAAAGCTTTTGCATATTATATCGAGCCACAGCATCGTGATGTTTTACAGCTTTATGCACCGG
AAGCTTTTAATTACATGGATAAATTTAACGAACAAGAAATAAATCTATCCTTGGAAGAACTTAAAGATCAACGGATGCTGTCAAGATATGAAAAA
TGGGAAAAGATAAAACAGCACTATCAACACTGGAGCG
>nmrc_003
TATGATATTAATCAAAGGTTGCAAGATACAGGAGGGTTAATTGATAGTCCGTCAATTAATCTTGATGTAAGAAAGCAGTATAAAAGGGATATTCA
AAATATTGATGCTTTATTACATCAATCCATTGGAAGTACCTTGTACAATAAAATTTATTTGTATGAAAATATGAATATCAATAACCTTACAGCAA
CCCTAGGTGCGGATTTAGTTGATTCCACTGATAATACTAAAATTAATAGAGGTATTTTCAATGAATTCAAAAAAAATTTCAAATATAGTATTTCT
AGTAACTATATGATTGTTGATATAAATGAAAGGCCTGCATTAGATAATGAGCGTTTGAAATGGAGAATCCAATTATCACCAGATACTCGAGCAGG
ATATTTAGAAAATGGAAAGCTTATATTACAAAGAAACATCGGTCTGGAAATAAAGGATGTACAAATAATTAAGCAATCCGAAAAAGAATATATAA
GGATTGATGCGAAAGTAGTGCCAAAGAGTAAAATAGATACAAAAATTCAAGAAGCACAGTTAAATATAAATCAGGAATGGAATAAAGCATTAGGG
TTACCAAAATATACAAAGCTTATTACATTCAACGTGCATAATAGATATGCATCCAATATTGTAGAAAGTGCTTATTTAATATTGAATGAATGGAA
AAATAATATTCAAAGTGATCTTATAAAAAAGGTAACAAATTACTTAGTTGATGGTAATGGAAGATTTGTTTTTACCGATATTACTCTCCCTAATA
TAGCTGAACAATATACACATCAAGATGAGATATATGAGCAAGTTCATTCAAAAGGGTTATATGTTCCAGAATCCCGTTCTATATTACTCCATGGA
CCTTCAAAAGGTGTAGAATTAAGGAATGATAGTGAGGGTTTTATACACGAATTTGGACATGCTGTGGATGATTATGCTGGATATCTATTAGATAA
GAACCAATCTGATTTAGTTACAAATTCTAAAAAATTCATTGATATTTTTAAGGAAGAAGGGAGTAATTTAACTTCGTATGGGAGAACAAATGAAG
CGGAATTTTTTGCAGAAGCCTTTAGGTTAATGCATTCTACGGACCATGCTGAACGTTTAAAAGTTCAAAAAAATGCTCCGAAAACTTTCCAATTT
ATTAACGATCAGATTAAGTTCATTATTAACTCATAAGTAATGTATTAAAAATTTTCAAATGGATTTAATAATAATAATAATAATAATAATAACGG
GACCAGCCATTATGAAGCAACTAATTCTAGACTTGATAGTAATTCTTGGGAAGCACCAGATAGTGTAAAAGGTGGCATTGCCAGAATGATATTTT
ATGTGTTCGTTAGATATGAAGGCAAAAACAATGATCCTGACCTAGAACTTAATGATAATGTTATTAATAATTTAATGCCTTTTATAGGAATATTA
GTAAAAGTGCCGAAAAGATCCTGTTGCAAAGCTTTTAAAGAACATATTATTCTATCAAGTGGCTGTATATTTTGTGTAATTTTCAATAAATTTTG
>nmrc_004
CCTTTTAATAATATGACATTATTATGTTCGTACATTGTGATGTTTTGTATAATAAACTGATTATATAATTCTAATGTATAATATTAATATGATTT
TAATATTAAGTTTTTAAGAACTTTCGCACACTATTTTTAATATCTAATTTAGGATTTTTATAGTATTTTTAAATTAAATTATAAAGAAATAAAAC
AAGAAATGTATAGATTCTTTAACCTCATATTTTCTATTAATTGATTTAGAGTATAGTCCCTATATCGTGATAAATTATATAAACTATATTCCTAA
ATTACCACTTTTATCTTTTCCAACAGAAGAATATTTTTGGCAGTTGCTTTCTCTTTCAATATATTTGCCACGCTCTTGTGGTCCTTATCAATTTC
AATCATACTATCTATTTACCGTATTAACTTTTGCTCATTAATGTAGTGTAATTAGTTCATTGGCGCTCGTTTCGTCTAATCTACTAACTACCTAT
AATAATTAAAGTCTTTTCGCAATATAGATTTCTGCTATGCTGTTAGTAAGACACCCTTACCTTAATACATAGAACCGTTTCTGCACCTGAACAGC
ACACCTTTACTACATTCCTTAAGGCATGAACCTTTATTTTGAGCATCAATATATTATAGATATCTTATTGAATGTGCAATTGTCCTCTAATTAAA
TTTTTCTGTGCTTTCTAAACTCCAGATACTTCGCACAATTGTATATAGAATCATTGGATGTTTCATTTTCTCTAAGTTTTTATGGATCCTACAAA
CAATCTCAAAGGATTATGATGATTTAGATTACTGTTTAAAACATACTCTCCTTGTATTTCTTACAAAAAGAGTATGTTTTAATTTTACTGTTTTT
TAGATAATTTAAAAATCACCTAGAATTACCTTATCCTATCTCATAGCCTTTTTTAGAAAAGATTAAAATTTTCTTGATCCCGTTGGTACTAGTAT
CCCCATTCTCACTAGGATTAATAATAGTGTTTTCTTTAGTAACAGCATATACATTTACCTTATAATTGGGATTACTTATATATAACGGTAATTTA
TCATTATATTTTTTAAAATCTATAAATGTTTTTCCATCTTGCCGTAAACTAGAAATATTCAACATATCATATCTGTCATTTATAACTTCTTTAAG
CCCTTCAGTATCTTCAATTTCTACAATATAACCTGATAATATTTTTCTTATATCCTTATCAATATTTAACAATAATCCCTCTGTTGACGAATTAA
TTACTTCTCTATGAGCCTCCTTAACTACTGACTCATCCGCCCCAA
>nmrc_005
AGTTGCGTTTAATTCCGCTAACTGATTCTTGATATTTTGAGATGTTTGTTGATCGAAATTAAAATCAAATTCGGTTATGTCTTTCCCTTGATATT
GTAAGTTTCCATTCGGTTCGTTAAATCCAAATGCTATTTTAAGGGCTTCTTTTAATGTCATATCCGGTTTAGTCGTTTCTAATGGATCACTAGGA
TTAACCGCCGCTATCCGCCTTTCTACCAGATTTAAATCTTTTCCATTAAAAATGATACGTGCAGTTGTTTCTTGAATTTGCGGTAACACTTCACT
CCAGTTCGAGCCTGTATCCACCCTCACTCTTCCATTTTCAAAATTGTATGTTGCTATATTCCCATATACTTGATCCGTATCTAATCTTAATTGTT
TCGTTTTTTTCTAACTCAAGAAATTGATTGTAATTCATTGTAATTGGAGTAGAACTGAAATCGTCTTGTGCATTTAATGCGATTGGCGCCAAGTTT
TTAGAAGGATAATAATTATTAGGTGCAAGTATTTGACTTAATTGGTTTTCCTTAGCTTTAATTGTCGCGAGTGTTTGATTTTTTCCTAACACTAA
CGAAGTCGTTGGTAACACGTTGTAGATTGGAGCCGTCCCAGTATTTACATATCTAATATTGGCATTTAATCTTGCTGTATCAGCGGTATTTAAAC

CCATTGTTTCAGCCCAAGTTCTTTCCCCTGCTAGAGATAGTGAATGATCAATTGCGACCGTACTTGAATTCGAATTACTAAATCCTGCAGATACA
CTCCCACCAATATCAAAGAACGACGCATGCACTTCTGCATTTCCATGTACTTCACTAGTATGTGTCCTACTTGTAGAAGTATTTTTACTTATTGT
TCTCGTTTGACTATCAGTATTCTGTGTGGATTGATCCTCATTTTTTGAGAGAATAATATTCTCCATATCTACATGTACAATCGGATAAGCTGCCA
CAAGGGGGTGTCTTGCCTCTGGTGATACATTCTTATCAATCCGTCCTGTAACCTTTTCGAAATCACTGTACGGATCAGAAGCCGTGCTCCATTTT
TCAGGAGATGATTTATATTTGGTTAATCCTTTCTTTTCATGAATATTAGAAATCCATGGTGAAAGAAAAGTTCTTTTATTTTTGACATCAACCGT
ATATCCTTCTACCTCTAATGAATCAGGGATTCCATCATTGTCACGGTCTGGAACCGTAGGTCCAGCACTTGTACTTCGCTTTTTTCTTGAGTTCG
AAGATTTTTGTTTTAATTCTGGCAATTGTAAGTTATCACTAGAAATCACTTCTTTTTTATTTTGAGAATCGGTCCAGTACAACTTGAAATCCAAT
CCTTTTTCAGTAGGATTTTCTCGTTGATATTGAATTTTTATTTGATATAATCTTCCTTTTTCTAATCTGATTTTGTTAGAATTAGAAGCTTTATT
AATCACTTCTTGGTCATCTACCCACATTGTTACATGATTATCAGCGGAAGTAGCAAATGTATATTCATCACTCTTCTTAACTTTGATAAATCCTG
ACCAAATAGCAGATTGAAAATATTGGTTTTCCGATGGAATATTTTCTAACTCAGAACTAGGAATAGATAAATCCCCTGTAGTAGAAGAGGTAACC
ACCATGGGTGCTTGAAAATTCAAATCACTAAAATAGTATCCTAGTAACCCCTGGGAACTTGATTCTGATTCATTTAATAACCGGTTCTCCTGTTT
AACTTCTGCCTGAATCACCTCTAAATTACCTGTGCTTGAAACTAATATCGTAGACAATGCCATTAATGGTATTAACACTTTTCGTTTTTTCATAT
ACGTTCTCCTTTTTGTATAAAATTAA
>nmrc_006
AAGGACCTTGAGTAATCAAAGTAAACTAAAAAATCTCCTTTTTGTATACTGATATTTAGAAAAAAATTACTTAGCAGCTTAGATATTTTTCCATT
TTACAAAGAACAATCCATTTATTACTCACCATTTATACTTATAGTAAACCCTAGAAAAACTAAAAACATCAGGAAGTACCCCTATTCAGGAAACT
TCTTATAAATATATATAGTTACTTCATTTTAAATAAAATCAAAAACAATAACAAAATAAAGCTTACAAAGTGGCTTTTTCTATATTATTGATACT
AACCATCTTGTAAATCTAGATAATACTGTATATTGGTGTAATCGCGTTCTGTAGGGATAGAATTAATACATATAATAGGGGTTGAACCTAAGTCA
AGAGGTATTGTTGAAATAATAAAGTCAGCATTAACATCGTGATCACTCTTTTAGCAAAAAAAACAGTTGAAATTTGTATTTTATCACCAAACCT
TTTTGCCAAAGTAGCAGCTAGATACTCTGCCCAACTTTCACTTTCTCCTGTGACAAGTAATACCTTTTTATAATTACTAGTATAACGAAGCCTAC
TTGCCTCTATATACATTGTAACTTTTGCAATTTCTTCAACAGATACATAGTCTGCTATCTCATTTTTTTGACAAATTCTGTGTATACTTCCTTT
ACTGCTGAGAAGGTTTCCGAATGTTCAGTTTGCATATACTGTATAGTTTGTTTTTGCGGCCTTTCAAACATACATAGGTATTGTAAATGATATAT
CGTTCTTTTAA
>nmrc_007
TATCGATATCCCTTGCTTTTAAGAATGTAAATTACTTTTATACATATACAATTCTAGACAGTATAAGTAGAGGTAAAATTAATTTCTTTACTCAT
TTTTGTTTGCAGAATGCAGTCAGAATCCCTGGTTTGTAATTATTAAGAACAAGGACATTTCCAATAAAAATATCCAATTTATATTCAAATTAATA
ATGTTACAATATTAATTAATATCACCTAATCATTAATATTTTTTATATCCTTAGTTATATGCGTTTTTATTTAGCAAGACATTTAACTGTTTTA
TAAAAAATTGCATTTCTGCCAAATAATATAATTATCATAAATAAAAATCAACATCCTTATTTGGTTATTATATAAATAGTCTCAGCCTATTACTT
GCGATACATTTATTATTCTTAGCATCTTAAATTCATAATGTTAAATCGCTTCCCCTCTAATCCTTTCCTAAAACTCTTAACGGCTTCGAGTTACC
ACCATTTCATATTTCAAATTAGCGATCTTTCTTAGTCAGTTTTTCTATTACTTCACGAGAAACTTTCTTCATTAATATTTCATTGTAAGAACCAG
TGTTAGGTGCGGTAACAGATATTATTGTTTGAACAATGATTTTTTACTTTTGCTTCATAATTTCCACTTCATATTACCACTATAATTTCTTTCAA
TTATGGTAAATAATAGATACCACAACTTAAATCTTAAATGCAATTCTCCTAAAATGTATTAGTTCTCAAACAAGGATTTTTTCTGCTTATGCAAG
CTGTTTTGGAAAAGATTCAGCAGTGTTTCCAATTTTCATTATAATTTATAGAGTTTTTCAATAACCTAAAAAAACTTCCTTTGATTAAGAAGTTT
TTTTGACTATTACAATAAAATTCATTTTTCAGCTAACATTCCCCATAAAAATATATATAAATTTTTTCTAATCACTCCTAAAGTCATTAATTTCA
AATACATTTCATTCTACGATAATTTCCTCATTGGTGTTACTTTCCAGTAATTAAGACACCGTCAAATCCGTTCTACTGTACTATAATGAAAATAT
TTTAACCACTGTTAAGATGCATATGCTAGCAATAGTTAGAGTTGGTTGTGATGTATCTTCACCAAGTAAAATCGGCACAGTTGAATATTATGTCC
AAATACAAAGAGAAGGTGAAATAAAAGGTAATGAACGTGTCTATACATTATTGACTTATGACAAAGAGGGAACAGAAAAGAAGGTCACATTTACT
AGTCAAAAGCCTAATAATGAGAAGCTAAAAGAAAATGCATTTTTACGTCTTTATATTAAACAAGAGGATAAAGAGAAAGCAGACATACCTGATAC
AGAAGTTAAATCCTATGAAGAGATACAGAAAGACGATTTACCAGCAAAAACAAAAGAACAATTAGGTGTTAAATAAAAGTATTGAGAATGACTTA
AAAGGGTTCTCATATATTAAGAGATACCCCTTTTAAGTTTATAAAATCAGAATATTATAAGATTTACTTGTTTATTGGAATTATAAATTTTCCA
CAATAAAAAAGAATCATAAATTTATTAACTCGAATAATTTTTCGTTTAAATTGTATAATACTCCTAAAATAAATGACGGCGTAACTTATTATCAC
AACAATTACCACTTCAATGGAAAAATCTGTAGAATGTAAGGAGGAACCCTCAAATAATTTTGAGTAAATACTAAGCTTATTTGAATCCTGAGGAT
GGAATTCACAATTGGTATAATTAAAAAATATATCGAAAAATCTAAATCACTTTGTATACTTAGGGGTTAGCCTGTAGATAATCACTAATCTTCTT
CTGCTCATCAGTCGTTAATGTTTCCGAACGTTTAAAGGTCCCCATTGTTACAAGTAATAAACCTACAATTAAAATCGGCATAATCCATCCAAAAA
CTTTTTTAACCATTTCTCCATTACCTCCTATTTATTTGATTTCCAAGTTCCATTTCTTCTGCCATCTCCAGCGCCGGTGATAACATTATCTCTCT
CGTCTTTAATTAAGGCCTGTACCCCACCAAAAAAGGCAGGGGATACTTTCTTCTTTACGTTCAATCCTTTTCTAGATAACTCATTTTTCACTTCT
GAACTTAGCTGAATCTCTGTATACGCTGTATTTTTTTCAAAAGTAAAACGGTATTCATTGATAATGTCTTGCAAGCTACCCTTACCATGCGTATA
TTTATCCAATATTGGGGTTAAAATTTGCGGAATACGGTTACCACCTGGTGACCCAATGCCGATCGTTTCCCCATCTTTCTTTAATACAGTGGGGG
CCATAAACGTTCGTGAACGTTTACCAGGTTCATAATTATTAAATCCCTCACTTCCAAAGTTCTGCAATTGATTATTTAAGAAGAACCCTGCTGTG
TACTTTCCTGTTCCAAAGAAATTGCTTAGTGTATTAGTTGAAGAGACAACCGTTCCATCTCTATCAATGATAACAAAATGCGTTGTGCTTTCATG
CTCTGCTTCCGAAAGCGCATCACCATTCTCATTCTTCATTGTTGATATATATTTGTCACTCACCATTTTATTTGGATCCATATTAACGTAATTTG
GATCCCCTAGGTTTTTCTTTCTATCTTGATAGGCAATCCTTGAAATCTCTTCCATTTTAGACATATAAGTTGCCGTATGATCTACATCTTTATAT
ACTTCTTTCTTTTCAGCTAATTTCAACATTTGTAATAAAGTAACTCCTGAAAAAGGTGGTGGAGCGGTATAAACATCATATCCCATGTAGTTACC
TTTTACTGGTTTACGTACCTCTACTTTATATCCTTTTATATCTTCTAACGATATTTTTGCAGTTTTACTGATTGCCCTAGCGACTCCTCCTTCAT
AAAAGCCTTTAGCCCCTTCTTTTTGAATCTTCTTTAAGGTTCTCGCTAAATCTGTCTGGATAAGTGTTTCTCCAGTTTCAATAGGTTCACCATTC
GGATAGAAGATACTTAGCTTATCAGAATAAATACGTGGCTTCGCAAGGTCTAATCGCATTGTTAAGGAATCATCTACCTTGAACCCTTTTTCCGC
ATAATTAATGGCTGGTTGTAATAACTCACCCATCGGTAATGAACCATAATTATCATGAATATACTCCATTCCAGCCACAAATCCGGGTACTCCAA
TATGTGGCTTTTGGTTTCCTGTAAAGTACGGAGTTGTTTCACGATAATCAATAAAGGTTTCTTTATCTTTAGATATAATGAGCATTCCACCGCCC
CCACCTATTCCTGAGGCATGCAGTTCTACAACGCCTAAAACATATGAGACCACAATCGCTGCATCTACTGCACTTCCACCGTTCTTTAATACTTT
CATACCTTCCTCAACCGCAAGGGGGTGAGAGGCACTCACTCCATAAGTTCCTTTATCACCCATACTATCAATTTTTTGCTTAACACTGTCTTTTA

TTTTATTGAAAGAACAGGATACCCCGATACCCCCCATTAAGCTGACTATCAAACAGAAAAGAATTATCTTCTTTCCCCATTTAAAGGAATTCAAG
TTGTTGTCTCCACTGATACTTGATTTTCTTTTTCTTGCTTATCTTGATGCTCTTGCTTTTCCCTCTTTTTCATTTTTTCAATAACATGTTTATGA
TTTAATTTGATTTCCAATTTATCATCTTTTTTACTCCATAGAGCACCCTTGGATGTATCTTTTGTTAATTGACGATACACACGATTTTTATCCAA
TGCACTGGTAACTGGTTTTGGTGATCCCTCTTGAATATTTAAAGGTACAACATCAAGAATTGCAGTACCATTATCACGTAAATGATATTGCACAA
GTGCACTATCTTTTGTTCTTGTCCATCCTTGGTCAAACACAAAGTTACCTAAACTATAGAAGATAATCCCTTGCTTATACACATCAAAAGATTGA
AGTACATGCGGATGGTGTCCCACAATAATATCTGCCCCTGCATCAACCATTGCTTTTGCTAAGGCTTCCTGTCTAGGACTCGGTTTATTATCGTA
TTCTTCCCCCCAGTGCGTATTTACTACGACAAGATCAGCATTACCTTTTTTAGGATCCTTTGCCTTACTAATTTGCTTAAGTAATACATCTGGGT
TCATACTTAACGAACCTGGTTGTTCTTTCGTTGCAATAGCTCCTGCTACAAATGCATCTGTAAATCCAAGAGTAGCAACCCTAACACCATTTACA
TTTTGATACACAATATTTTTTACATCTTTGAAATTTTCACCAGCACCCACATAGTCAAGATCAGCTTCTTTAAAGGCCTTTATTGTATCTTTAGT
TCCCTTAGCACCATAATCCGTCATATGGTTATTCGCCAAATTTAATACTGTAAATCCGGCTTCTTTTACTGCCTTAACTGTTTCTTCTTTTGCAC
TTAAGTGAATATTCTTATCTGCTTTTTGATAATTCTTTTTATCTTCTAACAAAACAGGATGTTCGAAATTCCCACTTACGTAATCTGAGTTTTTT
AAATATGGCGAAACATGACGAAAAACATAATCTGTACCGTAACGATTAACAATCTCTTTTACGTGACGTCCCATCATAATGTCACCAACCATCGT
CATCGTCAATTTTTCGTTCTCACGATGTTTTACTGGTGCTACTGCTTCTGTACGTTGTACCCATGTCGCAGCTATTAATATAACTGCGATAAGAG
GTAATACGATTGCTACATAACGAGGATTTTTTTTCTTGGTTTTCTTAATAAAGATCAGTAACTTTTCTTGAAATGTCAATTTTCGTCTCATTCTA
CCTCACCTTAAAATAAGTAATAAATATTCATGATTGCAAATGTTGCACCACTTAACAAAATTGTAGTTCCAATTGTTAATGGTAACCCTTGTCTT
TGAATTGTATTTGCAATTAATCCTGGAACAATAACTCCAATACCACGGAATTCAAAAATCTCAAATGGCATAACAGGATAACAATAATCAAATAA
AAGTTTTAAACAAATACCTGTAATTAGCGTTGCCGCAAATTTTCTACGGCCATATAAAATCATGAATCTTGAAACACCATACGTAACGATTACAT
ATGTTAAAATACTGATAAATAAAACAACCAACATAAATACGGGCTGATTAAAAACGAGTGCTAAATAACCAGGTACAACTAAACCTGCAGGTAAA
ATACCTGTTCTTTCTGTAAAAATAAGGCTCAGTGTAACTCCTAATACTAATGCAATATATAAATCTGATCCAAACATTCCTGTCCCTCCACTTAA
ATCACTTTTGCTTGCTTTTTGCCAATTTGTTCTTCCATAATCATATCGATTAATGGCTCAGCTGCACCATGAATATTACCCACTCCATATACAAT
CCGATTTTTTAAATATGGACGCATACGAGACATAATTTCACTTGTTGACCAGCCTTCTAAGTTCCAATACTCTTGCGTTGGAATATCTCCTTTTT
CAAAAGCACTTGTAATAGGTGCAGTCGTTTCTCCAATCGCAATAACTATTTCCGCTTTAATATATGGCAAAACATCCCTAGCAAACTGCTCAGTA
CGATCAACGCGGTCAGGGCGGCAATTCATAATTACAATTGGAGCTAGATTACTATATCCAAAATCATCCACACGTTCCCAAATACGTAATGTTGA
TGAGGGATCATTCGCTGCAAAACCATTTACGAAGAACGCAGGCTTAGATTGGTCAGCAAAACGTGTAATTCTCATTGCTCCTGGATCCGGATGAG
CATTCAACATACCACGGAATGCTGTTTCCTCATCAATCCCAAGAGCCTCTGCTACCGCTAAAGCAAGCGATGCATTATCTGGGAAGACCATGTAA
TCAAATTTTCGTAAGAATTCTTCTGAAATTCTAGAATTATCCGCAACAATCACTTTTGTATTTCTCTCTTCTGCAACCTCTTTAAAGTAATCCAA
GTATTCACTTTCAATAGTGACTAAATGTCCATTATATGGAATGGTAGCAGTGAAAGCTTCAGCT
>nmrc_008
TCTCCGATATTACGTCCTTGAAACTCACTCATCCCTACATCAGCAATCGCTTTTTCTACCTTTTCCTTATCGTTTTTCGTGAAAAAGCGAAACAA
TCCTTTTTTCGAAACGAGTCCCATTGACACAACTTCAAACACAGTTGCCGGAAAACCAGAATTAAAGCTATTTGCCTTTTGCGACACATAACCAA
CTTTGTTCCATTCTTTAAACTTCTTACTATCAACACCAAACAAACGAATGCTTCCCTGTTTTGGCTTTAAAACGCCTAATAAACATTTTAGCAAA
GTTGATTTCCCAGAACCATTCGGGCCAACTAAACCTAAAAAAGCTCCCTTCGGAACTTGCAAATTAATATCTTCTAACACATTTCGATCTTCATA
TCGAAATGACAATCCTTCTATTTCTAATATATTATTCATAGCATCTCACCTATTTTAATTCAGAATGATTCCGATTTATATCTATTTGAATTATA
GTACAGCTTATTATAGTTGTAAACCAATCTGCCTAAAATATATCTACTTTTTCAAGAAACCTTACTATTATAACAAAAAGCAGAGAAAAATTCTC
TGCTTTCGAAAAATATTCCTATAAAATGAAACCTTAATGAGATGAGGCCATCCCCTACCGATTGGTAAAGGCTTTTACTGGATAATTTTTTTAGT
GAATAGCCGATTTAAACTTACCACCATTTGTCTCTTGTGTACTCATAATCGTAACAAATGCATTCGCATCAATTTCATACACAATTGATTTCAGC
TTTGTCACTTCCAAACGCGTTACAACTGCATATATTACTTCTTTTTCTTTATCCGTGTAACCGCCTTTCGCTACAAGCTTTGTAGTTCCACGACC
AAGACGATGTAAAATCGCATTTGATACTTCCTCATATTGATCTGATACAATTAAAACTGCTTTCGTTTCATCTAAACCTTGAATGACTGTATCAA
TTGTTTTGAACGCAATATAGTACGTCATAACAGAATACATAGCTTGTTCAACACCAAATACAAATGCTGCCCACGCAAAAATGAATAAGTTCACA
AACATTACAAATTCGCCAACGGAGAAAGGTAATTTTTTCGTTAATAAAATACCCATAATTTCTGTTCCGTCCATTGATCCACCATGACGAATAAC
GAGCCCTACACCAAGCCCTAAAATAAGACCACCAAACACTGTCGCTAAAATTGGCTCTGTTGTAAATGGCGGAACAGCATGTAACGTTGATTCAA
TAAACGCTAAAGCTACAATACCGAACGCCGAAGATAGCATGAACGTTTTTCCTATTTGCTTATAACCTGAATACATAAATGGGATGTTGAGAATA
ACGACTAAAGTAGAGAAGCTTAACCACCAAATATTAGGAGTAAGATAATCTAGTATAAGAGAAATACCAATAATTCCACCGTCAATAATTTTATT
CGGCATTAAAAATAATTCAATCGCTACCGCTGCACATGCTGCCCCAAAAATAATCATAACTAAACGATAAATAAGATGGATAACACTTTCTTTTC
GATGTTGCTTTTGCTCCATAAATCCTCCCCATACTCTTTTTCTATAGTTTTGTCTCTTTATTATAACATACGCTTCTTTTTGTCATGAAAAAAGA
AACTCTATACGAATATGTACAAGCTACGTACTCATATATTTCGATAGAAGAACGTGTGAAGGAGGATAAAACGATGAACCTCATTAAACAACTTG
TAAATAAAAAATTAAATCATATTTCTACAAAAGATTTATTGAAATATAGTAAAGAATATGAAGTTCCAATTACGACTGCACAAGCTGATCAAATT
GTTGTACTTATGAAAGGAAAGAATATTAACATTTATGATAATGACGAACGACTAGAGCTCTTAAAACAAATAGCAAAAGTAACCTCCCCTGCTAC
TGCCCAACAAGTAAATACTTTATTTCAGCAACTACTAAAATAAGGAGGGGATATCCCCTCCTTATTGCGCTTTAATTTTTTCAAGAAGTCCTTCA
TCAAAAGTACCATTTTTCAGCATTTCGATTTCTAATTTATATGGCGGCTTCTTATCTTTTTTATCTTCACCTACATATGGCGTTTCAAGAATTTT
TGGTACGTGCGTTAACTGTGGATGATGTACAATATGATGCAATGCTTTATAACCGATATGACCGAAACCAATATTTTCATGACGGTCTTTTCCTG
CGCCGCGTACATTTTTACTATCATTAATATGAAGTACTTGTAAACGATCGATACCAACAATCTTATCAAATTCGTTTAATACACCGTCAAAGTTA
TTTACAATGTCATATCCTGCATCGTGCGTATGACATGTATCAAAGCATACTGATAGTTTTTCATTATATTTTACGCCATCAATAATTTTTGCAAT
TTCCTCGAAACTACGGCCGCATTCTGTTCCTTTTCCTGCCATCGTTTCTAACGCAATGTTAACAGTCTGATCTGGCGTTAACACTTCATTAAGTC
CTTTAATAATCTGTTGAATACCAGCATCCGCTCCTGCACCAACGTGCGCACCTGGGTGAAGAACGATTTGTTTCGCCACACCTAATGCTGATGTT
CTCTCAATTTCCATACGAAGGAAATCTACACCTAATTGGAATGTTTCTGGCTTCGTCGTATTTCCGACATTAATAATATATGGCGCATGTACGAT
AATCTCTTCAATACCGTTTTGTTCCATATGTTTTCTTCCTGCTTCTATGTTCAATTCTTCAATTGGTTTTCTTCTTGTATTTTGCGGTGCACCTG
TATAAATCATAAACGTTGTTGCACCGTATGAAACAGCCTCTTCACTTGCTGCTAATAACATTTTCTTCCCGCTCATGGAAACATGAGATCCAATC
TTTAACATACAATCACCTCTTCAATATACAATAGATATAATGATAGCATAATTTGTAGAAATATGTAGTGAATTGTTTGTTCACAAAAAACGCAC
TACATAAATTTATACGACTATTTAAACAGACTACACTCTTCATTTACATTATCTAGATGAAGCAAAACTTTAATCAATATTTTCTATCCATATTG

ATTAAAGCTCTCA
>nmrc_009
CAACCATACATGCAGCATCATCCCGAGCAAATGATCCCTCCTCAAATGTATGAATCAAACGAAACGCGCGGCGGTGCAGCAACTACAGCAGCATC
AAGTAGCGGCATCGGTAGTTTTTTTTCGAATTTAATTTCGAATCCAACTAATATGATAAATAATATCGAAAAAGTATCACAAGTCGTTCAATCTG
TAAGCCCTGTCGTCGAACAGTACGGTCCCATTATGCGTAACCTACCAAGCATCGTTAAAATCCTCACCTCTGGAAAAAGTACGGAAGAAAATCCA
ACCGAAGATCAAACTGAAGACCTAACAGAAAAGGTTGAAGTAGCAACTCCACCTCCTCCACAAAAAAAAAGAAAAAGAAAAAAATGGTGATTGAG
CCAGTTATAGAAAAAGAAGTGCGCGAGGAGCCTGTTCAAAAAATAGCAACAAAACCAAAACTATATGTGTAACAATCCTTTGTTTTCTATCCACT
CCTCCTTTATAATGTAAAAGACTATGCACAAAAGTATCCCTTGTTTAGAAGGAGAGGATTACTCATATGAAAATTGTTAAAATTTCCCCTCGTGG
TTATTGCTACGGTGTTGTGGACGCGATGGTTATTGCACGTAACGCCGCATTAGATACATCATTACCAAGACCTATTTATATTTTAGGTATGATTG
TTCACAACAAACATGTAACAGATGCATTCGAAGAAGATGGTATCATTACATTAGACGGTCCAAGTCGATTAGACATTTTAGATAAAATCGATTCT
GGTACTGTTATTTTCACTGCACACGGTGTTTCTCCAGAAGTTAAACAGCGTGCAAAAGAAAAAGGTTTAACAACCATCGATGCCACTTGCCCAGA
TGTTACAAAAACACATGACCTTATTGAAGCAAAGAAAGCTGAAGGTTACCATGTCATTTATATCGGCAAAAAAAATCATCCAGAACCGGAAGGCG
CAGTTGGGATCGCACCTGATATCGTTCATCTTATCGAAAGAGCCGATGATTTAAAAACATTAGAAATTCCAACGGATAAAATTTTAGTTACAAAT
CAAACAACGATGAGTCAATGGGATGTGCAACATTTAATGGAGGACATTCAGAAAAAAATTCCCAACAGCAGAGTTCCATAAGGAAATTTGTTTAGC
AACTCAAGTTCGCCAAGAAGCTGTTGCCAAACAAGCTGATGTTGCCGACTTAACAATTGTTGTTGGTGATCCGAAAAGTAACAACTCAAACCGTT
TAGCACAAGTATCACAAGAAATCGCTGGTACGAAGGCATACCGCGTTGCAGACGTAAGTGAGATTAAATTAGAATGGCTACAGGGTGTAGAAAAC
GTAGCTGTTACAGCAGGTGCTTCTACTCCAACACCAATTACAAAAGAGGTTATCGCTTTCTTAGAGCAATATGACCCAATGAATCCCGCTACATG
GGAGAGAGTTCGAAAAGTACCGTTACAAAAAATATTACCTCGTGTAAAAGTGAAAAAAGAACAATAATAAAAACCGTTGCCTATATGAGCAACGG
TTTTTATTTTTCTTATACAAATGTAAATGGATCTGTATGTAACTGCGAAGCATGAATGTTTACATTAAGTTTCTTTGCATCCACTTTTTCTTGTA
ATTGCTTTTGTACACCTTGCTTCATTACCTTTTCAACGTTATGTCCTGGGTCAACTATATTTAAACCGAGCATCATCGCATCATGAGCAACATGA
TAATACATGTCCCCCGTTACATATACATCTGCTCCTTTAAATTTAGCTTGATTGATGTATTTGTTACCATCGCCACCAAGTACAGCTACTTTGCG
CACTTTATCATCTAATTTCCCAACAACTCGCGCACCCTTTACATCTAATGACTTCTTTACATGTTCCGCAAATTGTCCAAGTGTCATTTCTTCTT
GTAAATATCCTATTTTTCCAAGCCCTAATGTTTCACCTTTGTTATCAAGTGGATACACATCATATGCTACTTCTTCATATGGATGTGCCGTTACC
ATTGCTTTAATTACTTTTCGCTGTAATGAAGCTGGAATAATCGTTTCGATTCGCACTTCTTCCACGCGTTCTAACTGCCCAGTTTCCCCGATATA
AGGATTTGTTCCCTCT>nmrc_010
TTTCATTTGCGCAAAAAAATGTACGTCAGGGAGGGAAGGACGCTCGTCCTAAATGAATGGAAAAAAGTTGGTATGTTGTCCATACTTATTCTGGA
TATGAAAATAAAGTAAAAGCAAACCTAGAGAAACGTGTAGAATCAATGGGTATGCAAGATAAAATTTTCCGTGTTGTTGTCCCAGAAGAAGTAGA
AGTAGAAATGAAAAACGGTAAAGAAAAATTAATGAAAAGAAAAGTGTTCCCAGGTTATGTATTAGTAGAATTAATCATGACTGATGACTCTTGGT
ATGTTGTACGTAACACGCCGGGTGTAACTGGGTTCGTTGGCTCTTCTGGTTCTGGATCTAAACCATCACCTCTATTAGAAGAGGAAGTTGTTACC
ATTATGAAACATATGGGAATGGACAACGAAGTGGTTGATTTCGACTTTGAACTTCATGAGACAGTACGTGTAAATGAGGGACCATTCGCAGATTA
TACAGGTGCTATCGAAGAAATTGATGTGGAGAAGAAGAAGGTTAGCGTACTTGTGGACATGTTTGGTCGCGAGACTCCAGTTGAACTTGACTTCC
ATCAAATTGAAAAATTATAAAATGAAACTTGAAATGAATTGTAAAAAGTGATAATATCTTTTAAGTCAGTACGTCTTCGTTATCGGAGACGTTTT
TTGAAAGATTTTATCCTTACAGATAAAATATGACGTGGGAGGGCAAATCACTGTCCAATTGACCACATCACGGACTTAAGGAGGTGTGTCTCGTG
GCTAAAAAGGTAATTAAAATGGTAAAACTTCAAATTCCTGCAGGTAAAGCTAACCCAGCTCCACCAGTTGGTCCAGCATTAGGACAAGCAGGTGT
TAACATCATGGGCTTCTGTAAAGAGTTTAACGCTCGTACAGCAGATCAAGCTGGTCTTATCATCCCTGTTGAAATTACGGTATTTGAGGACCGTT
CATTCACTTTCATTACTAAAACTCCTCCTGCTGCTGTTCTTCTTAAGAAAGTAGCTGGTATTGAGTCTGGTTCTGGTGAACCAAACCGTAATAAA
GTGGCAACTGTTAAGCGTGATAAAGTACGCGAAATCGCTGAAACTAAAATGCCTGACCTAAACGCTGCTAGCGTAGAAGCTGCAATGCGTATGGT
TGAAGGTACTGCACGCAGTATGGGCATCGTTATCGAAGACTAATTCGATTTGTTTTTAAAAAAGGTTGCGGGTCTGGAATTCCAATTCGCAACC
TTTATTATCGTAAATGATTATCGTTTTTAAATAAATGGATGGCGCGCATCCTCAGGTTATACCTGAAAATAAGCGTAAACGTGGGAGGTTATTCC
GCTAAAACCACATTCGAGGAGGAAATAAAAATGGCTAAAAGAGGTAAAAAGTACGTAGAAGCTGCAAAGCTTGTTGATCGTGCAGCTGCTTACTC
TGCAACAGAAGCAGTAGAATTAGTAAAGAAAACAAACACAGCTAAATTTGATGCAACTGTAGAAGCTGCATTCCGTTTAGGTGTTGACCCTAAGA
AAGCTGACCAACAAATCCGTGGTGCAGTTGTTCTTCCACACGGTACTGGTAAAGTACAACGTGTATTAGTATTCGCTAAAGGTGAAAAAGCTAAA
GAAGCTGAAGCTGCTGGAGCTGACTTCGTAGGCGATGCTGATTACATCGGTAAAATCCAACAAGGTTGGTTCGATTTCGATGTAGTAGTAGCAAC
TCCTGACATGATGGGTGAAGTTGGTAAACTTGGTCGCGTATTAGGACCTAAAGGTTTAATGCCAAACCCTAAAACTGGAACAGTTACTTTCGATG
TAACTAAAGCTGTTAACGAAATCAAAGCTGGTAAAGTTGAATACCGCGTTGATAAAGCTGGTAACATCCACGTTCCAATCGGTAAAGTATCTTTC
GAAGATGCTAAATTAGTAGAAAACTTCAGAACAATTGCTGACACTTTACAAAAAGTTAAGCCAGCTGCTGCAAAAGGTACTTACATGAAGAACGT
AACAGTTGCTTCTACAATGGGACCTGGCGTACGTGTAGACGTTTCTACATTAGCGTAAATTTTGGAAGTTGACTTCATAAAGAAGTTTTTATATA
ATCATTTATGTTGTGAATTTAAATAGTGTACCGTAGACAGTAGGTGTCAATAGACTTAATTTCCTACCTAGGTGTTAATATACGAAGCGGAATTT
TTTTCTGTGACTATATGCCTCCATGTCTACAAGTTGGGCATGGAGGTTTTAGTGCACTTTCGGTACATCTTCTATATAATCTACAGGAGGTGTA
ATAACATGAGCAAAGTAATCGAAACTAAACAACAAGTTGTAACTGAAATCGCGGACAAACTTCGCGCTAGTAAATCTACAATCGTTGTTGACTAC
CGTGGTTTAACAGTTTCTGAAGCAACAGAATTACGTAAGCAATTACGTGAAGCTGGCGTTGAGTTCAAAGTTTACAAAAACTCTCTAACTCGTCG
TGCTGCAGAATCTGCTGAAATGGCTGAGTTAAATGAATTCTTAACAGGACCAAACGCAATCGCGTTCAGTAACGAGGATGTAGTTGCTCCTGCGA
AAGTATTAAACGACTTCGCTAAAGATCATGAAGCTTTAGAAATTAAAGCGGGCGTAATCGAAGGTAAACTTGTAACACTTGATGAGGTTAAAGCT
ATCGCTACTCTTCCATCACGTGAAGGCTTACTTTCTATGCTTCTTAGCGTTCTTCAAGCTCCAATCCGTAACCTTGCACTTGCTACTAAAGCAGT
TGCAGACCAAAAGGAAGAGCAAGGCGCTTAATTTTTTAAAAGATAATTACGTATTATCGATAAAACAATACAAACCTATTTAAGGGAGGATATTT
ACAATGACTAAAGAACAAATCATTGAAGCAGTTAAATCTATGACTGTATTAGAACTTAACGACTTAGTAAAAGCTATCGAGGAAGAATTCGGCGT
AACTGCTGCTGCTCCTGTAGCTGTTGCTGGTGGCGCTGGAGAAGCTGCTGCTGAGAAAACTGAATTTGATGTGGAACTAACTAGCGCTGGTGCAC
AAAAAATCAAAGTTATCAAAGTTGTTCGTGAAATCACTGGTCTTGGCTTAAAAGAAGCTAAAGAATTAGTTGACAACACTCCAAAAGTAATCAAA
GAAGCTGCTGCTAAAGAAGAAGCTGAAGAAATCAAAGCTAAACTTGAAGAAGTTGGCGCTGCTGTAGAAGTTAAGTAATTAACTTTTGATGCTTT
AAAAAAAGCTCGCTCTCATGCGAGCTTTTTTTAACTGTAAAGAAAAGAGGTGGCCATATGGCAGACCATTATTTTTCTAACGACCCTTCTAGTAA

AAGTGATCGTAAGCGATGGGAATTTACGCTTCGTGGATCTCGATTTACTTTCTTATCTGACCGTGGGGTGTTCTCGAAAAACGAAGTGGACTTTG
GTTCTCGTCTTTTAATTGAAGCGTTTCAAGTGCCAGATATTAAAGGTGATATATTAGACGTAGGTTGTGGATATGGACCGATTGGTTTATCGTTG
GCGAAAGAGTTTCAAGACCGTAAAGTTCACATGGTGGATGTGAATGAAAGGGCGCTTGAGCTTGCGAAAGAAAATGCCGCTAACAATAGAATTGG
AAATGTGCACATTTTTCAAAGTAGCGTCTACGAAAACGTAGATGGTATGTATGCTGCTATTCTATCTAATCCTCCAATTCGTGCAGGTAAAGATA
TCGTGCATGAGATTTTAGAAAAAGCTGTAGAGCATTTAGTTCCAGGTGGAGAGTTGTGGATTGTTATTCAAAAGAAACAAGGTGCACCATCTGCA
CTGAAGAAACTAGAAGAAGTGTTTTCTGAAGTCGAAGTTGTAGAAAAGAAAAAAGGATATTATATCATAAAATCAAAAAAACGTTGACGGTTATT
TTTGGCTATGTTAACATTATACAATGCCAATATATGATTTTCTGCGTTGAGAAAGATGTATATTTTTGTTTCTCTTGGAAAAGATAGTAAAATCA
GCAGATTATGAAACAGAATGATGGTTTTCTTATAGAAGCCATTTTTCTTTTTTGAGCAGGTAGAAAGACTCAACGTATTTATCTTTAAGAGAAAA
AAGACTACGCTAAATAGCAGTAGTTGTATTTATTTGTGATTTTGCACAAATTTTTTGTGCATTTATAATACTCATGATTTGAGGGGTGAAGCAGT
TGACAGGTCAACTAGTTCAATACGGACGCCACCGCCAACGAAGAAGTTATGCCCGTATTAGTGAAGTATTAGAGTTACCAAATCTTATCGAAATT
CAAACCTCTTCTTATCAGTGGTTTCTTGATGAGGGTTTGCGAGAAATGTTCCAAGACATTTCTCCGATTGAAGACTTTACGGGAAATCTATCGCT
TGAATTTATCGACTACAGCTTAGGTGAACCTAAATACTCTGTAGACGAATGCAAAGAGCGTGATGTGACGTATGCAGCACCACTTCGTGTAAAAG
TGCGTCTAATCAACAAGGAAACTGGTGAAGTAAAAGAACAAGATGTGTTCATGGGAGATTTCCCACTCATGACAGAGACTGGAACATTCGTAATT
AACGGTGCAGAACGTGTTATCGTTTCCCAGTTAGTTCGCTCTCCAAGCGTATACTATAGTGGCAAAGTGGATAAAAACGGAAAACGTGGTTTTAC
TGCTACTGTAATTCCAAACCGCGGAGCTTGGTTAGAGTATGAGACAGATGCTAAGGATGTTGTATATGTGCGTATTGACCGTACGCGTAAACTTC
CTGTAACTGTTTTGTTACGCGCATTAGGGTTTGGCTCTGATCAAGAAATCACCGAGCTTTTAGGTGATAACGAATACTTAAGCAACACATTAGAA
AAAGACAACACAGATAGCACAGAAAAAGCATTGCTTGAAATTTATGAGCGTCTACGTCCTGGTGAACCACCAACAGTAGAAAATGCCAAGAGCTT
ACTTGTGTCTCGTTTCTTCGATCCAAAGCGCTATGATTTAGCAAATGTAGGTCGCTACAAGATCAACAAGAAGTTACACATTAAAAACAGATTGT
TTAACCAACGTTTAGCTGAAACATTAGTAGATCCAGAAACTGGTGAAATTTTAGCGGCAGAAGGAACAATCTTAGATCGTCGTACACTTGATCGC
ATTTTACCTTACTTAGAGAAAAACATTGGATTCAAAACAGCGAAACCAATGGGTGGAGTGGTAGAAGGTGATGTTGAGCTGCAATCTATTAAGAT
TTATGCTCCTGAGTCAGAAGGCGAGCGCGTAATTAATGTAATTGGTAATGCAAACATTACTCGTGATGTAAAACACATCACACCAGGTGATATCC
TTGCTTCTATCAGTTACTTCTTCAACTTACTATACAAAGTAGGGGATACAGATGATATCGACCACTTAGGAAACCGTCGTCTGCGTTCTGTTGGA
GAACTATTACAAAATCAATTCCGTATCGGTCTTTCTCGTATGGAACGTGTTGTTCGTGAGAGAATGTCGATCCAAGATACAAATGCAATTACACC
ACAGGCATTAATTAATATTCGTCCTGTTATTGCAGCTATTAAAGAGTTCTTCGGAAGTTCTCAGTTATCTCAGTTCATGGACCAAACAAATCCAT
TAGCAGAGTTAACTCACAAACGAAGATTATCTGCATTAGGACCTGGTGGTTTAACGCGTGAGCGCGCAGGCTTTGAAGTACGTGACGTTCACTAC
TCTCACTATGGTCGTATGTGTCCGATTGAAACACCAGAGGGACCAAACATCGGTTTGATTAACTCATTATCTTCGTTCGCGAAAGTAAATGAGTT
TGGTTTCATTGAAACACCATACCGTCGTGTTGACCCAGAAACTGGTCTTGTAACAGGGCATGTTGATTATTTAACAGCAGATGAAGAAGATAATT
ATGTTGTAGCCCAAGCGAATATGAAATTATCTGAAGAAGGAGAATTCCTTGATGAAGATATCGTAGCTCGTTTCCGTGGTGAAAACATTGTCACA
AATAAAGAACGCATCGACTACATGGATGTATCACCAAAACAAGTAGTGTCGGCAGCGACAGCTTGTATTCCGTTCTTAGAAAACGATGACTC
>nmrc_011
TTATAGGCTCTGGAGCTGTAATGTTGAGCGGGCCATCAATTTCTTTTTTGTGTATGATGAAATCAATCATGGAAGCGACATCGTCTATATGAATC
CATGATAACCATTGCTTTCCAGATCCAACTGTACCTCCGATATAGAATTGATAGGGAAGTAGCATTTTGGGAAGGGCTCCTCCGTCTGGACCTAA
TATGACTCCGAATCGTGCATAGATTGTTCGTATTCCGAGAGAGCGTGCTTTAGATGCTTCTTGTTCCCATGAATATACTGTATTTGCTAAAAAGT
CATTTCCGGGAGTCACATGCTGTTCTGTAAAAGACTCGGTTTCAGACGTTCCATAGTATCCAATTGCACTCGCGTTAATAAATGTATGTGGTTTT
GCAGGGAGTGCCTGCAATTGTTTAATGAGTCCTTTTGTTGTTTGAATTCTACTGTTTAAAATGATTTTCTTTTGTTTCTTTGTCCATCTACTATT
AATAGACTCTCCAGCTAGATTAATAACTACATCAATAGAAGAGAGAGGGAAGGTTTGTAAATCTGGTGTCCATTGCACATACTGAAGGTTAGGGT
GAGAAGTTTCAGTAGTTTTTTTTCTTGTGAGAATGTAAACCGTATATCCTTTTTGAATAAAAAAAGTAGAAAGGTATGTGCCGATAAAGCCGGTA
CCACCAGAAATTGCGATTTTCACAATTATTTCCTCCTCATTATATATATTCTTGAAAAGCAAAAACGTTCCTCGACTATGTTAAAATAATAGAGA
GGTGAGAGTATGGCTGTCATTACAAAAATAGAAGTGCAAAAACGATCGAAAGAACGGTTTAATATTTATATCGATAAAGGTCAAGGTGAAGAATA
CGGATTTAGTGTGAACGAAGTAATCTTAATAAAGCACGGATTACAAAAGGGCTTAGAAATTGATGAAATAGCGTTAGGAAATATTTTGTACAATG
AAGAGGTACAAAAAGCATATTTACAAGCAATCTCCTATTTATCCTATCAAATGAGAACGAAACTAGAAATAGAAGATTTCTTACGAAAAAAAGAA
GTGGGACAAGCCATCATCTCTGAAGTCGTTTCGAAATTATTACATGACCGATATATTAATGATAAAGAGTATGCTATTTTATATACGAGAACGCA
AAGTAATGTGAATCGAAAAGGTCCAACGGTTATTAAAAGAGAGTTGTTAAATAAAGGTGTTCAGGATCTAATTATTATGCATAGTTTACAAGAAT
ATACGAAGGAAAAGCAAATTGAGAATGCTTTAATTCTTATAGAAAAGAAGAAAAAGTCTTATCAAAAGCATTCCTTTTTACAAATGAAACTAAAG
TTAGATGAAATGCTTGTTCGTAAAGGATATTCTAGAGATGTGATTCAAATTTGTTTGGAAGAATTGAAAGACGAAAAAGATGACGAAAAGCAACA
AGAAGCGTTACACTATCATGGGAACAAATACTATGAGAAATATAAGAAGTATGATGGATGGACGTTTGAAAATAAGATGAAACAAGCGTTATATC
GTAAAGGATTCTCTATTGATGAGATAGAGATATTTTTGCAAATGAAACGTGAAGAGGGATGAGGGAATAAAATGAATATGCCAAAACGCTACAGT
GAAATGACACCATATGAGCTAAGGGAAGAAATTGGGGTTTTGAAAGAGCAAGCAATAAAAGCTGAGCAACTTGGAATTGTAAATGAGTTCGATGT
ATTAATGCGAAAGATAGCAATGGCTCGTGCTTATATGACTGACATAAATAAATTCCATATTGGTGAGACGTATGAATTAGTAGAAGAACCTGGTA
TATTATTTGAAATTACGTATTTCAATGGGGTATTTGCTTGGGGACATAAGCAAAATGATAATGAAGAGATTGGAATACCGATTTCTTTATTACAA
GAAAAATAAAAACCAGAGAGCGAATATCTCTCTGGTTTTCATCTTTGGTAGGCGTTACAGGAGATAGGAGATTCTAAAATTGGCGTTTTTTACAT
TCATTCGCCAATATAATGAGAGACTGGGTTTGTTGCGTCTGTCCGTTAACTTGCGCTTTTGCATGTTTCGGACGAGTCCACGTATGGTTAAATAA
TTCAGAACGACTATCTAAGCGATCACGGTAGCTCATCTTTATCACCTCGTGTAGGAAGTTGAAGTTAAATTACTTTGTACTACTCTTCCTGCTGA
TTTGCAGCACGCATACGTTCTTGTGGGTGCGTGTTAATTGTGCCGTTAGGTCGCTTCGAAGCGAAGCGTGATTTCGCTTTCGGTTGACCGTCGAT
TTTGCTGTTGTTTTTTGACTCAGACCAAAATTCGGCTTGTTTACCCATTGCGAACGCCCTCCTCATCATCAGTTGATACCTCTTGAAGAAGTATC
AATTATAGAATGTGCAAAATAGAAGAAACTATCCTTTAGAAATGAAGGGATAATTAGGTAAAGGAGATTAGAACATGAATGATATTTATGAAGCA
TTAACGAAAGAACTATTAGAGAAAAATGACAAACTTTCTTATGCACAAGCTCGTGCGTGGGTTGAATTACTGTGGGAAGATTTTCAAACAACTTA
TGCGAAATCAGGTCGTTATCAAGGAGAAGAAATGACTGAACAAGTGGTGCGATCATGGATTAATAATCATGGAGGACGCCTTCATGAAATGCGTA
CAAATAATCCGAAATATAGCCATCTAATCAATCAAGAAGATCATTTGAA
>nmrc_012

GGAAGTAACTCCAGTTTTTTTCGAAGTTTTTCTTCACTAAAAATCCATCCTGTATAGGAACTAATAATATTTAAATTATGATCGAGTTGAACGAT
TGCAACGAATGGATAATAATCTTTACTGCGATATCGCAAATCAATGAATCGAACTTCATAATAATGATCATAATCGAAAATATCCCAGCGATACA
CAGGAGAGAAAGATAAAAACGCTGAGATGTTTTCATCTTGTTTAGCTGCGCGCATAATAGCATTATCTGGGAACGGTACCCGATCGAACTTGTCA
TATATCATGATATTACCACGGTGCCATCTCGCAACGTAGTAATAATCAGCCGTAACGACTGCTAAATGGTAATGATAAAATCGGTAAGAAGGAGA
AATGATAATCTTTTCGACATTTTTGAAACGTTTGTGTACAACGCTTCTTATATTTCTGTGCATAATAATCCGTCCGATGTAGTAAATAATCATTA
ATATATACGCTGCTAAGGCAGTATACCCTTTATGGGAACCTACGAGCATACAAGCAATTGCGAGTATATGGATAAAGAAAATGACGGTATCAAAT
GTATTAATGATGCCGAGTGCTACCCATTTTTTTGTGAAGGGCCGTAATGCTTGTGTACCGTAGGCATTGAAAATATCTACAAAGACGTGAAGAAA
GACCGCAATAAATGACCAAAGTAGTAGATGAAGATATGGAGCGTCTGAAAAGAAGGCGAAAGAGATGCCACTTATAAGGAACGACCAAAGAATTA
CTGCAGGAATGGAATGAGTAATCCCGCGATGATTTCTTATATATTTAGCGTTATTACGCAATTTTAAAACTGTATCGATGTCAGGAATATTGGAA
CCAGCAATTGTAGCAAGCATAACAGCTTGAGGACCAATATCACTTTGTGCTATGGCTGGATCTAACGTGGCCAAACTGCCTAGAGTAACGCCCAT
AACAAGGTGAGTGGCTGTGTCCATAGAAATCCACCTCCGTTTTTTATTAATGTAACTCTTTTTATTCGATACCTCAAGTCCTTTGCCTTCTATTT
CCTATGTCATCTTACTTAAATTTTTTCCTAAAATCTTTATAATAGTACTTATATGCAAAAAAATGAGGGGGATCAAGTTTGACACTTGAAATATT
AAATAACTTTAACATAGAGCAGTTTCAAAATGATTTAATTGGTTGGTTTGAAAAAGAGCAACGTGACTTACCGTGGCGTAAAAATAAAGATCCAT
ACCGCGTTTGGGTTTCGGAAATTATGTTGCAGCAAACGAGGGTAGAAGCTGTAAAACCATATTACGCGAATTTTATGGGGAAGTTTCCTACTCTA
GAAGCTTTGGCCAATGCTGATGATGAAGAAGTGTTAAAAGCATGGGAAGGTTTAGGGTATTATTCTAGAGCGCGAAATTTACATGCGGCTGTAAA
AGAAGTAAAAGAAGTATATGGCGGGATTGTGCCAAGTGATGTAAAGAAGATTGAGAAATTAAAAGGAGTCGGCCCCTATACAAAAGGTGCTATTT
TAAGTATCGCATATGGCATACCAGAGCCGGCAGTTGACGGAAATGTTGTGCGTGTATTGTCTCGTATTTTATCAGTATGGGATGACATTGCAAAA
CCGAAAACTAGAAAAGTGTTTGAAGAGATTGTGCGTGAAATTATTTCGGCTGAAAATCCATCTTATTTTAATCAAGGTTTGATGGAATTAGGAGC
ACTGATTTGTATTCCTAAAAATCCAGCATGCTTACTTTGTCCTGTTCGTGAGCATTGCAGGGGATATGCTGAAGGTGTTCAAAAGGAATTACCAG
TAAAAAGTAAAGCGAAAGCTCCTACGATGGTACCGATTGTTGCAGGAGTACTTCAAACGGAAGATGGTCGTTACGTCATTAATAAACGTCCAAGT
ACCGGTTTATTAGCTAATATGTGGGAGTTTCCGAATGTTGAACTTGGCGAAGGGATTCGTAATCAGAAGGAACAGCTTATAGATTATATGAAGGA
AAAATTTGAGCTTTCGATTTCTATTGAAGAATACGCAATGAATGTACAACATACGTTTACACATCGTACTTGGGATATATTCGTATTTTATGGAA
AAGTAACTGGTGATATTGTTGAAACAGATACATTGAAATTTGTATCGAAAGAAGCATTTGAGCAGTTACCTTTCTCTAAATCGCATCGTACGATT
TATGAGAATTGTGTTGAGAAAATTACAATGCAATAAACAAAAAAAGTGTTCCCAGGTTGGGAACACTTTTTTAATCGTAAGAAATTTTTGTCCCA
TGAGTCCAACTAGCTTCACCTTCTTGTAAGCCACCTCTTGATTCAATTTCTATTACAATTTCTTTGTAAATACTTTGACCTTCCGCGTTTAAATA
AGGCAAAATCTGTTGTAATGAATGATGAAAATAAGCTAATTCATCTTCTTTCCATTCGTTTTTTGAAACCATTGTTAGTTCAGTCATATCGCGTC
CTATATACATATTCCCACCTCCATTTTCTATAGTTTGAATGAGTAACAAGAGATATATGCGGAATTTTTTCAAAAGAAAATTACGGATAGCTTCT
AGTTGAATTGGTTACATTATTGATTGTGGAGGTGAGAAAGATGAGTAAAAAACAACAAGGTTATAACAAGGCAA

Note that the first and last twelve bases of each fragment are not capable of being queried on the RA.
