# Supplementary material for: Genotyping of Bacillus cereus Strains by Microarray-Based Resequencing
Source: PLoS One. 2008 Jul 2;3(7):e2513. doi: 10.1371/journal.pone.0002513 (PMC2438477; doi:10.1371/journal.pone.0002513)
Supplement: Table S4 — Proportion bases called (QS 30) for each B. anthracis molecule (0.04 MB PDF) [file pone.0002513.s006.pdf]

	pXO1	*pXO2*	*Chromosome*
BAN_001	0.82	0.17	0.92
BAN_002	0.99	0.18	0.92
BAN_003	0.97	0.95	0.96
BAN_004	0.74	0.95	0.88
BCE_001	0.13	0.15	0.68
BCE_002	0.15	0.15	0.76
BCE_003	0.13	0.15	0.75
BCE_004	0.14	0.14	0.81
BCE_005	0.15	0.16	0.82
BCE_006	0.11	0.14	0.76
BCE_007	0.71	0.13	0.71
BCE_008	0.08	0.11	0.28
BCE_012	0.17	0.17	0.54
BCE_013	0.19	0.19	0.82
BCE_014	0.18	0.16	0.63
BCE_015	0.17	0.15	0.23
BCE_016	0.18	0.20	0.72
BCE_017	0.15	0.17	0.78
BCE_018	0.18	0.18	0.85
BCE_019	0.24	0.18	0.26
BCE_020	0.17	0.13	0.22
BCE_021	0.13	0.15	0.74
BCE_022	0.18	0.17	0.74
BCE_023	0.18	0.19	0.71
BCE_024	0.11	0.12	0.25
BCE_025	0.12	0.12	0.43
BCE_026	0.16	0.18	0.89
BCE_027	0.10	0.11	0.72
BCE_028	0.04	0.05	0.44
BCE_029	0.42	0.48	0.48
BCE_030	0.65	0.59	0.44
BMG_001	0.12	0.13	0.31
BMY_001	0.19	0.17	0.69
BMY_002	0.18	0.16	0.57
BMY_003	0.19	0.18	0.62
BMY_004	0.14	0.13	0.36
BSU_001	0.24	0.23	0.27
BSU_002	0.23	0.23	0.27
BTU_001	0.20	0.17	0.87
BTU_002	0.19	0.19	0.68
BTU_003	0.25	0.20	0.28
BTU_004	0.19	0.19	0.82
BTU_005	0.15	0.15	0.68

The values are shaded dark gray if over 0.6, light gray if between 0.4 and 0.599.
