# Supplementary material for: Genotyping of Bacillus cereus Strains by Microarray-Based Resequencing
Source: PLoS One. 2008 Jul 2;3(7):e2513. doi: 10.1371/journal.pone.0002513 (PMC2438477; doi:10.1371/journal.pone.0002513)
Supplement: Table S5 — Bacillus resequencing results. (0.05 MB PDF) [file pone.0002513.s007.pdf]

ABACUS Quality Score Threshold

Bacillus Strains	10	15	20	25	30	35	40	45	50	55	60
**BAN_001**	95.03%	94.41%	93.82%	92.92%	91.98%	90.83%	89.64%	88.48%	86.95%	85.33%	83.44%
**BAN_002**	95.13%	94.41%	93.67%	92.75%	91.66%	90.54%	89.32%	88.03%	86.55%	85.18%	83.59%
**BAN_003**	96.36%	96.35%	96.29%	96.22%	96.14%	96.03%	95.82%	95.55%	95.24%	94.78%	94.14%
**BAN_004**	95.01%	93.74%	91.93%	89.89%	87.65%	85.34%	82.48%	79.41%	76.09%	72.37%	68.11%
**BTU_001**	91.06%	90.22%	89.35%	88.33%	87.37%	86.28%	85.16%	84.15%	83.02%	81.68%	80.53%
**BTU_002**	80.43%	77.36%	74.21%	71.24%	68.40%	65.40%	62.79%	59.96%	57.56%	55.22%	52.71%
**BTU_003**	37.31%	35.03%	32.99%	30.74%	28.36%	26.12%	24.01%	22.14%	20.57%	19.18%	17.88%
**BTU_004**	88.51%	86.95%	85.37%	83.61%	81.97%	80.19%	78.40%	76.72%	75.05%	73.43%	71.70%
**BTU_005**	80.03%	76.69%	73.43%	70.75%	68.16%	65.67%	63.32%	61.15%	58.80%	56.61%	54.58%
**BCE_001**	79.59%	76.03%	73.03%	70.34%	67.63%	65.20%	62.89%	60.90%	58.76%	56.74%	54.71%
**BCE_002**	83.42%	81.46%	79.62%	77.63%	75.79%	73.89%	71.92%	70.00%	67.92%	65.90%	63.81%
**BCE_003**	84.76%	81.97%	79.57%	77.08%	74.82%	72.56%	70.01%	67.62%	65.04%	62.56%	60.10%
**BCE_004**	87.88%	86.22%	84.51%	82.78%	81.03%	79.36%	77.79%	76.25%	74.64%	72.97%	71.23%
**BCE_005**	88.66%	86.97%	85.27%	83.55%	81.74%	80.05%	78.42%	76.71%	74.93%	73.20%	71.45%
**BCE_006**	85.13%	82.71%	80.33%	77.87%	75.53%	73.18%	70.74%	68.25%	65.68%	63.17%	60.42%
**BCE_007**	81.83%	78.80%	76.23%	73.82%	71.45%	68.88%	66.51%	63.84%	61.10%	58.24%	55.29%
**BCE_008**	54.20%	46.43%	39.64%	33.55%	28.42%	23.84%	20.17%	16.76%	13.92%	11.52%	9.47%
**BCE_012**	70.04%	65.58%	61.36%	57.39%	53.74%	50.48%	47.44%	44.40%	41.56%	38.87%	36.07%
**BCE_013**	88.36%	86.87%	85.23%	83.64%	82.08%	80.49%	78.64%	76.80%	75.40%	73.58%	71.91%
**BCE_014**	76.76%	73.35%	69.96%	66.55%	63.36%	60.22%	57.28%	54.60%	52.00%	49.37%	46.96%
**BCE_015**	43.28%	36.75%	31.28%	26.62%	22.90%	19.72%	17.19%	14.89%	13.06%	11.43%	10.01%
**BCE_016**	82.03%	79.66%	77.05%	74.57%	72.25%	69.84%	67.63%	65.79%	63.81%	61.95%	59.98%
**BCE_017**	85.36%	83.56%	81.78%	79.96%	77.88%	75.86%	73.68%	71.37%	69.04%	66.75%	64.20%
**BCE_018**	90.63%	89.55%	88.25%	86.79%	85.21%	83.62%	82.07%	80.61%	78.92%	77.18%	75.48%
**BCE_019**	35.40%	33.33%	30.85%	28.60%	26.30%	24.16%	22.07%	20.35%	19.02%	17.56%	16.33%
**BCE_022**	82.57%	80.30%	78.10%	75.84%	73.61%	71.57%	69.56%	67.49%	65.61%	63.85%	62.01%
**BCE_023**	81.80%	79.02%	76.28%	73.29%	70.65%	67.98%	65.77%	63.54%	61.50%	59.36%	57.37%
**BCE_024**	50.42%	41.93%	35.54%	29.66%	25.13%	21.55%	18.43%	15.86%	13.72%	12.06%	10.46%
**BCE_025**	63.84%	58.47%	53.52%	48.14%	43.02%	38.73%	34.89%	31.14%	27.37%	24.00%	20.92%
**BCE_026**	93.03%	92.06%	91.02%	89.82%	88.61%	87.17%	85.81%	84.36%	82.99%	81.38%	79.83%
**BCE_027**	82.39%	79.59%	76.90%	74.36%	71.99%	69.47%	67.10%	64.69%	62.20%	59.51%	56.72%
**BCE_028**	58.90%	53.96%	50.21%	46.96%	43.96%	41.30%	38.71%	36.41%	34.18%	31.85%	29.84%
**BCE_029**	76.80%	70.29%	63.08%	55.51%	47.76%	40.43%	33.47%	27.20%	21.44%	16.00%	12.04%
**BCE_030**	74.87%	67.70%	59.97%	51.71%	44.47%	37.29%	30.64%	24.36%	19.10%	14.72%	11.02%
**BMG_001**	53.64%	46.71%	40.76%	35.76%	31.24%	27.56%	24.08%	20.86%	18.01%	15.59%	13.34%
**BMY_001**	81.10%	77.87%	74.62%	71.55%	68.53%	65.87%	63.16%	60.61%	57.97%	55.28%	52.74%
**BMY_002**	74.64%	69.64%	65.15%	60.91%	56.92%	53.52%	50.07%	47.02%	43.96%	41.40%	38.82%
**BMY_003**	76.33%	72.47%	68.95%	65.57%	62.38%	59.12%	56.29%	53.94%	51.15%	48.91%	46.60%
**BMY_004**	58.39%	51.62%	45.92%	40.53%	36.32%	32.26%	28.90%	25.47%	22.35%	19.57%	17.24%
**BSU_001**	36.56%	34.34%	32.02%	29.41%	26.85%	24.72%	22.89%	21.08%	19.59%	18.28%	17.06%
**BSU_002**	34.79%	32.90%	30.85%	28.79%	26.85%	24.81%	22.92%	21.28%	19.76%	18.42%	17.23%

Bacillus Resequencing Results. The percent of bases called for different strains at a given ABACUS threshold is shown. Colors reflect *B. cereus* clade identity as shown in Figure 2. Purple (*B. cereus* Clade 1), Orange (*B.cereus* Clade 2), Blue (*B. cereus* Clade 3), Green (Other Bacillus species).
